# Effects of Web-Based and Mobile Self-Care Support in Addition to Standard Care in Patients After Radical Prostatectomy: Randomized Controlled Trial

**DOI:** 10.2196/44320

**Published:** 2023-09-06

**Authors:** Camilla Wennerberg, Amanda Hellström, Kristina Schildmeijer, Mirjam Ekstedt

**Affiliations:** 1 Department of Health and Caring Sciences Linnaeus University Kalmar Sweden; 2 Department of Surgery Region Kalmar County Kalmar Sweden; 3 Department of Learning, Management, Informatics and Ethics Karolinska Institutet Stockholm Sweden

**Keywords:** eHealth, linear mixed model, prostatic neoplasms, radical prostatectomy, randomized controlled trial, self-care, telemedicine, mobile health, mHealth, prostate cancer, sexual dysfunction, urinary incontinence, web-based, pelvic exercise, physical activity

## Abstract

**Background:**

Prostate cancer is a common form of cancer that is often treated with radical prostatectomy, which can leave patients with urinary incontinence and sexual dysfunction. Self-care (pelvic floor muscle exercises and physical activity) is recommended to reduce the side effects. As more and more men are living in the aftermath of treatment, effective rehabilitation support is warranted. Digital self-care support has the potential to improve patient outcomes, but it has rarely been evaluated longitudinally in randomized controlled trials. Therefore, we developed and evaluated the effects of digital self-care support (electronic Patient Activation in Treatment at Home [ePATH]) on prostate-specific symptoms.

**Objective:**

This study aimed to investigate the effects of web-based and mobile self-care support on urinary continence, sexual function, and self-care, compared with standard care, at 1, 3, 6, and 12 months after radical prostatectomy.

**Methods:**

A multicenter randomized controlled trial with 2 study arms was conducted, with the longitudinal effects of additional digital self-care support (ePATH) compared with those of standard care alone. ePATH was designed based on the self-determination theory to strengthen patients’ activation in self-care through nurse-assisted individualized modules. Men planned for radical prostatectomy at 3 county hospitals in southern Sweden were included offline and randomly assigned to the intervention or control group. The effects of ePATH were evaluated for 1 year after surgery using self-assessed questionnaires. Linear mixed models and ordinal regression analyses were performed.

**Results:**

This study included 170 men (85 in each group) from January 2018 to December 2019. The participants in the intervention and control groups did not differ in their demographic characteristics. In the intervention group, 64% (53/83) of the participants used ePATH, but the use declined over time. The linear mixed model showed no substantial differences between the groups in urinary continence (β=−5.60; *P*=.09; 95% CI −12.15 to −0.96) or sexual function (β=−.12; *P*=.97; 95% CI −7.05 to −6.81). Participants in the intervention and control groups did not differ in physical activity (odds ratio 1.16, 95% CI 0.71-1.89; *P*=.57) or pelvic floor muscle exercises (odds ratio 1.51, 95% CI 0.86-2.66; *P*=.15).

**Conclusions:**

ePATH did not affect postoperative side effects or self-care but reflected how this support may work in typical clinical conditions. To complement standard rehabilitation, digital self-care support must be adapted to the context and individual preferences for use and effect.

**Trial Registration:**

ISRCTN Registry ISRCTN18055968; https://www.isrctn.com/ISRCTN18055968

**International Registered Report Identifier (IRRID):**

RR2-10.2196/11625

## Introduction

### Background

Prostate cancer is one of the most common forms of cancer worldwide. The survival rate is >93% thanks to improved care [1], meaning that a growing number of men are living with the consequences of treatment. One of the most common treatments is radical prostatectomy, which can have side effects that decrease the quality of life [2]. The most commonly reported side effects of radical prostatectomy are urinary incontinence and sexual dysfunction [3]. The psychological impact of a prostate cancer diagnosis and its treatment, with uncertainty and worries about the future [4,5], may impact recovery and engagement in self-care. Participation and engagement in self-care increase the likelihood of living life as desired [6].

Self-care recommendations for patients with prostate cancer focus on pelvic floor muscle exercises and physical activity but also include recommendations on tobacco cessation, penile rehabilitation, and limited alcohol consumption [7]. Sexual function can benefit from pelvic floor muscle exercises [8] and physical activity, which can increase feelings of masculinity [9]. Sexual rehabilitation includes pharmacological treatments for erectile function, partner engagement, and processing of psychological aspects, making self-care recommendations multifaceted [10]. Physical activity has been found to reduce incontinence [11] and cancer-specific fatigue while increasing cancer-specific quality of life, fitness, and body strength [12]. Pelvic floor muscle exercises are recommended, as they have been shown to shorten the time of recovery from urinary incontinence postoperatively [13]. Although research indicates that adherence is crucial for the efficacy of pelvic floor muscle exercises [14], it may be difficult to mobilize and maintain motivation and self-care behaviors over a long time, resulting in increased symptoms. Men with prostate cancer describe that they need to change and adapt their lifestyle so that self-care can fit into everyday life [15] and they need easily accessible and individual support for self-care throughout rehabilitation [16]. A recent review and meta-analysis showed that the most significant benefits of pelvic floor muscle exercises seemed to be achieved under the guidance and supervision of a therapist, compared with when using only verbal instructions. The availability of therapists may vary, and alternative routes to access support could benefit patients [17].

Systematic reviews show that interventions to increase physical activity in prostate cancer rehabilitation can have positive effects; however, more research on optimal delivery methods to reach patients throughout their rehabilitation has been suggested [18,19]. Furthermore, internet-based programs for psychosocial support show positive effects on psychological aspects but not on health-related quality of life [20,21]. Programs often reveal low engagement in the interventions [22], and there is a scarcity of research on long-term effects [23]. Achieving behavior change requires programs that go beyond providing information and instruct on why a change is needed and how it can be made [19]. Web-based interventions to support patients with prostate cancer differ in design, and no consensus has been reached on the best way to engage patients in long-term self-care for symptom relief [24]. Furthermore, the interventions currently offered tend not to be adaptable to the differing needs of men with prostate cancer across the recovery trajectory [22,25]. Although some interventions show improved symptom burden in patients with prostate cancer in the first months after surgery [18-21], to our knowledge, the long-term effects have only been sparsely evaluated in randomized controlled trials.

Providing web-based support for a range of problems in cancer rehabilitation could be a way to meet the increasing number of patients where they usually go for information and support [26], thereby increasing accessibility. Digital self-care support called electronic Patient Activation in Treatment at Home (ePATH) is a web-based and mobile app [27,28] accessible to patients for 1 year after radical prostatectomy. It offers cohesive support for self-care, focusing on self-care to reduce the most common problems after surgery (urinary incontinence and sexual dysfunction).

### Objective

In this study, we compared the effect of additional ePATH support with the effect of standard care alone on postoperative complications and adherence to self-care after radical prostatectomy over a 1-year period. The specific aims were to investigate the effects of the ePATH intervention on (1) urinary continence and (2) sexual function and adherence to self-care recommendations in (3) pelvic floor muscle exercises and (4) physical activity.

## Methods

### Study Design

This multicenter block-randomized controlled trial with 2 study arms had a longitudinal design with follow-up measures at 1, 3, 6, and 12 months after surgery. The study was conducted in routine clinical practice to strengthen external validity and increase the possibility of implementation at a larger scale [29]. The study was designed in accordance with the Medical Research Council’s framework for the evaluation of complex interventions [30,31], and a study protocol has previously been published [32]. The study followed the CONSORT-EHEALTH (Consolidated Standards of Reporting Trials of Electronic and Mobile Health Applications and Online Telehealth; version 1.6) [33]. The trial was registered in the International Standard Randomized Controlled Trial registry (ClinicalTrials.gov NCT18055968) and International Registered Report Identifier (10.2196/11625).

### Participants and Setting

**Figure 1:**
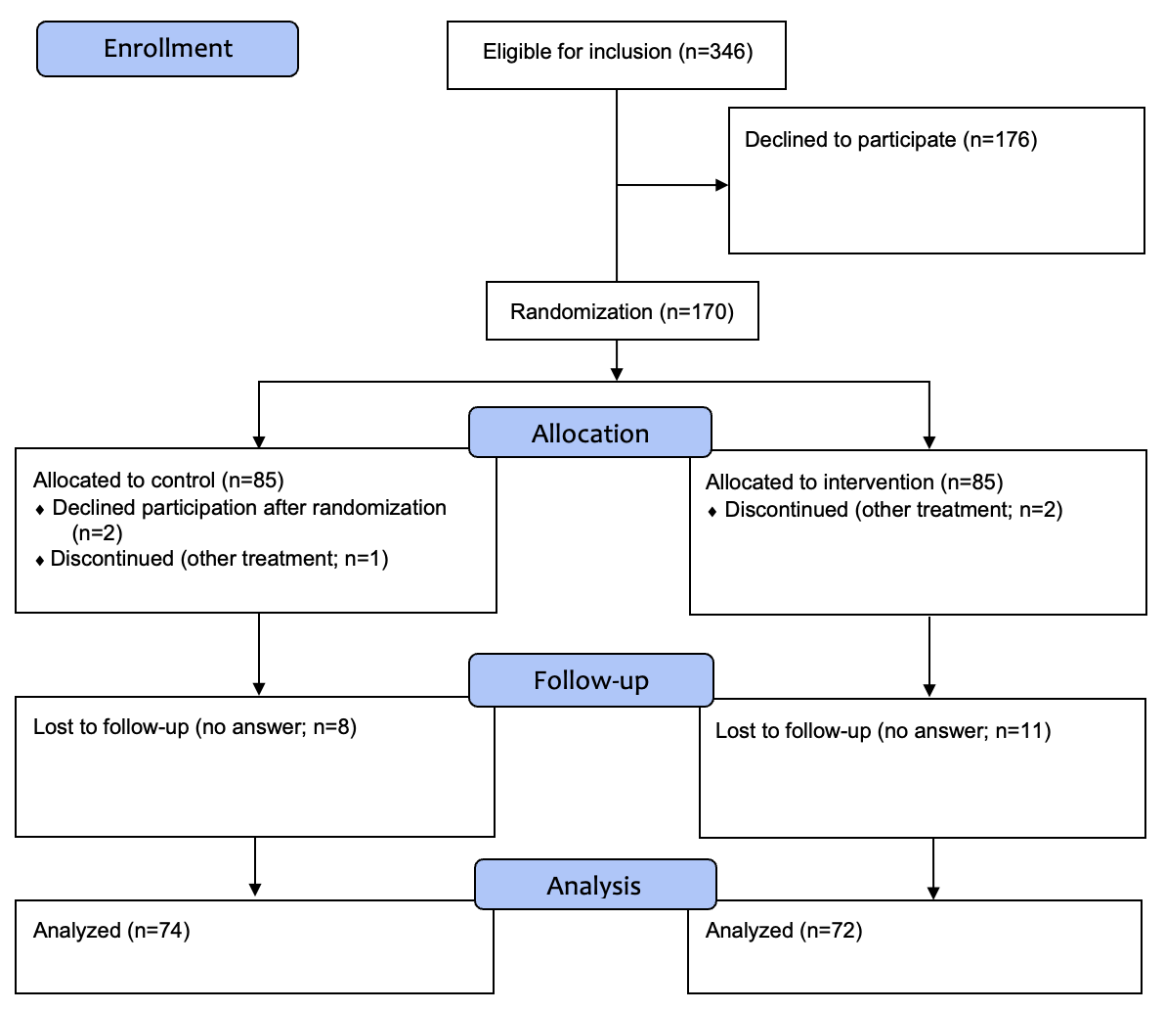
CONSORT (Consolidated Standards of Reporting Trials) flow diagram. The intervention group and control group allocated 85 men each. Five men withdrew from participation because of other treatment or without giving a reason. No answers were received from 19 men, who were considered lost to follow-up.

A total of 170 men (Figure 1) from 3 urology departments at county hospitals in southern Sweden participated in this study. The 3 hospitals were situated in regions with approximately 94,000 to 360,000 citizens. Each of the 3 sites performed 45 to 125 radical prostatectomies annually (2020). The care organizations at the 3 sites differed somewhat, meaning that the inclusion process varied slightly, but all the included clinics used the national standardized care trajectory. One site provided preoperative information in groups and regular postoperative appointments with a clinical sexologist. Another site provided individual preoperative information, postoperative sexual medicine counseling by a urotherapist, and a series of group seminars after treatment. The third site offered preoperative individual information and postoperative counseling by a sexual medicine counselor. All patients could contact their cancer nurse specialist or urotherapist when needed.

### Sample Size

The sample size was calculated with a 2-sided 5% significance level and power of 80% based on the sample size table and suggested end point provided by developers of the Expanded Prostate Cancer Index Composite (EPIC) [34]. As recommended by the developers of the EPIC, to show clinical importance, the effect size was set at 0.5 [35], and a medium effect [36,37] and 4 domains of EPIC were used. This required a sample size of 114 patients per group, given a dropout rate of 25% [28].

### Recruitment

Recruitment began in January 2018 and was completed in December 2019. Data collection was completed in January 2021. All men scheduled for radical prostatectomy who met the inclusion criteria at the 3 hospitals were eligible for inclusion. They received preoperative written information about the study and a manual for ePATH from their cancer nurse specialist in conjunction with the treatment decision. Approximately a week after receiving written information, the men were asked verbally by the cancer nurse specialist about participation; if they chose to participate, a signed consent form was sent by post to the researchers. Inclusion took place every alternate week, during which new participants received the baseline survey by one of the researchers (AH).

The inclusion criteria were having intermediate- or high-risk localized prostate cancer, being treated with radical prostatectomy, being aged >18 years, being able to speak Swedish, having a facility for secure mobile log-in, being computer literate (assessed by each man himself), and having an active email address. The exclusion criterion was having a cognitive impairment, to the extent that the patient would not be able to participate in the intervention fully (assessed by the cancer nurse specialist from medical records and personal communication).

### Intervention

#### The ePATH Intervention

The intervention group received access to ePATH through the web or mobile app in addition to standard care [7]. The ePATH intervention was developed by the research group to address the need for self-care support that has been identified during rehabilitation after radical prostatectomy [32,38]. ePATH was tested for quality and usability in a collaborative approach with end-user groups to address various needs for self-care support [27,28]. Minor bugs in the system were fixed during the evaluation process, but no changes were made to the content. The ePATH intervention is theory driven (self-determination theory) and based on the assumption that autonomy, competence, and relatedness foster intrinsic motivation and enhance engagement in self-care [39,40]. ePATH serves to increase the patient’s activation by improving the knowledge, skills, and confidence to manage self-care as well as creating the necessary support for adopting the desired self-care behavior into daily life and maintaining it over time.

The cancer nurse specialists at the 3 sites received information and instructions on how to administer and introduce ePATH through meetings with the research group at the beginning of the study. One researcher (CW) was the primary contact person for the cancer nurse specialists during the inclusion of patients if questions arose or if technical support was needed.

The cancer nurse specialists tailored the information and support in ePATH into interactive self-care modules based on each patient’s needs. For example, if a patient used tobacco, a module concerning tobacco cessation was added, and other modules could be adjusted depending on whether a patient was physically active or not preoperatively, to fit the patient’s goals. Self-care in ePATH focuses primarily on pelvic floor muscle exercises (Kegel: contractions and relaxations of the pelvic floor muscles), which are recommended 3 times per day, and physical activity, including endurance and resistance training. To further support sexual rehabilitation, patients were provided with supplementary information on changes that could occur in sexual function after radical prostatectomy and on available pharmacological treatments for erectile dysfunction. The participants could use and personalize their account to fit individual circumstances and preferences in whatever way they preferred, including times of day or days of the week to perform exercises. The participants were free to use the ePATH account as they felt appropriate [28].

One module provided individualized information about the diagnosis, treatment, and rehabilitation. Another module explained why self-care was warranted. Patients had the possibility to register self-care completed, rate self-care efforts (eg, intensity of physical activities), and set goals for self-care. Patients could also receive reminders on self-care as notifications through the mobile app if they chose to activate that feature. One module provided information on how to assess health and evaluate self-care. Patients could see graphs of performed self-care in relation to symptoms experienced (eg, pelvic floor muscle exercises in relation to urinary continence over time). ePATH also included a function for messaging the cancer nurse specialists for support and guidance through a secure pathway. The cancer nurse specialists read and answered patient messages daily (asynchronously) but did not check patient engagement in ePATH.

#### Control Condition

The control group received care in accordance with the standardized cancer care trajectory in Sweden (standard care). The national strategy states that each patient with cancer should be offered a personal cancer nurse specialist for psychosocial support and coordinating care. All men scheduled for radical prostatectomy received written information on self-care as well as verbal information about rehabilitation, pre- and postoperatively, in conjunction with regular contact with health care. In accordance with the standardized care trajectory, the cancer nurse specialists contacted men in conjunction with the regular prostate-specific antigen checkups, usually performed at 3- or 6-month intervals, asking about overall well-being and side effects of treatment [7].

### Randomization and Blinding

Three block randomization lists (1 list per site) were created by an independent statistician using Microsoft Excel to ensure an even distribution in the intervention and control groups between sites. The predetermined randomization lists were kept in sealed, sequentially numbered envelopes, consecutively opened by CW to reveal the allocation to the intervention or control group. CW created ePATH accounts for the men in the intervention group, and the cancer nurse specialists individualized the information and functions. All men were notified via email (from CW) regarding their randomization into either the intervention or control group, approximately 1 week after completing the baseline questionnaire. Those in the intervention group also received a message in ePATH from their cancer nurse specialist, informing them when their account was ready to use.

Both groups answered web questionnaires (paper was not an option) at baseline and at 1, 3, 6, and 12 months after radical prostatectomy. For each follow-up, 2 reminders were sent by email (within 10 days). Questionnaire responses were processed by CW. The nature of the intervention meant that there was no possibility of blinding the intervention researcher (CW), cancer nurse specialists, or participating men.

### Measures

#### Characteristics of the Participants

Self-reported demographic data (age, education, household income, and marital status) were obtained at baseline. Data on the Gleason score (used to evaluate cancer severity), length of hospital stay, complications, and nerve-sparing surgery [7] were retrieved from the medical records by the cancer nurse specialist once the patient provided informed consent.

#### Primary Outcomes

Two domains of the EPIC were used to study the primary outcomes (urinary continence and sexual function). This is a validated comprehensive questionnaire for examining patient function and bother after prostate cancer treatment, including surgery, radiotherapy, and hormonal therapy. The questionnaire contains 26 questions encompassing the domains *Urinary Incontinence*, *Urinary Irritative, Bowel, Sexual,* and *Hormonal*. Here, the *Urinary Incontinence* and *Sexual* domains were chosen because they were relevant for patients after radical prostatectomy. The other domains mainly focus on function and bother after radiotherapy and hormonal treatment [41].

The *Urinary Incontinence* domain contains 4 questions measuring urinary continence. The *Sexual* domain includes 6 questions concerning sexual function after prostate cancer treatment. For both these domains, answers are given on a Likert scale with 4 or 5 levels and then converted to a 0 to 100 score. The total score for each domain was calculated by adding the scores for each question and dividing it by the number of questions. Higher scores represent better urinary continence (less urinary incontinence) and better sexual function (better erectile and orgasmic function and overall satisfaction). The scores on these 2 domains were calculated 1, 3, 6, and 12 months after surgery. At baseline, single questions measuring urinary continence and sexual function from the respective domains of the EPIC were used.

#### Secondary Outcomes

Physical activity was assessed using the Saltin-Grimby Physical Activity Level Scale [42,43] at all time points. Participants rated their level and frequency of physical activity per week on a 4-point scale: 1=sedentary, 2=some physical activity, 3=regular physical activity and training, and 4=regular hard physical training.

Pelvic floor muscle exercises were assessed using a single item on the frequency of postoperative pelvic floor muscle exercises. Participants rated their performance on pelvic floor muscle exercises as 0=never, 1=once a day, 2=2 times a day, 3=3 times a day, or 4=>3 times a day.

#### Use of ePATH

Log data were retrieved from the ePATH server to investigate use. Men in the intervention group were categorized as ePATH users if they had logged into ePATH more than once or registered self-care in ePATH. Those who had logged in once or not at all were categorized as nonusers.

### Statistical Analyses

Data were analyzed using SPSS software (version 26; IBM Corp) for Windows [44]. Analysis of the missing data showed that 19 participants had not provided data on any of the outcome measures. Not all individuals need to be included in an intention-to-treat analysis, as the accuracy of the analysis is based on whether its assumptions are valid [45]. The analysis used here can be referred to as a modified intention-to-treat analysis [46]. We excluded participants without any data, resulting in 146 participants (74 in the control group and 72 in the intervention group) being included in the final analysis (Figure 1). An analysis of the missing data patterns for the remaining participants showed missing values at the individual level over time for approximately 15% of the outcome measures. Therefore, we imputed the data using multiple imputations with predictive mean matching [47]. Five imputation rounds were performed, and the pooled values of these imputations are presented. An analysis of sensitivity was performed, comparing the final models with unimputed data [45,48].

Descriptive statistics, such as percentage distributions, were used to describe the participant characteristics and baseline data. Means and SDs were used to describe the normally distributed continuous variables. To identify differences between groups (intervention or control), Pearson chi-square test was used for nominal variables, the Mann-Whitney *U* test for ordinal variables, and the independent 2-tailed Student *t* test for continuous variables. A 2-sided significance level of <.05 was used for all statistical tests. Normal distribution was assessed based on visual evaluations of histograms and plots. Individual trajectories were plotted on a simple line graph for the sample to obtain an overview of the variation at baseline and the development over time.

We conducted 2 different multivariate analyses to investigate the longitudinal effects of treatment on primary and secondary outcome variables.

Linear mixed models were used to investigate the difference between the intervention and control groups in primary outcome measures, in a sequence of 4 models, and to assess the mean score differences over time for continuous variables [49]. We applied fixed effects of time and group using an unstructured correlation structure with 4 repeated measures (1, 3, 6, and 12 months postoperatively).

To investigate the longitudinal effects of the ePATH intervention on the secondary outcomes (physical activity and pelvic floor muscle exercises), ordinal regression with generalized estimating equations was used, with 4 repeated measures (1, 3, 6, and 12 months postoperatively).

Interactions between time and group were tested in all models (both linear mixed models and generalized estimating equations). Interactions indicated differing trajectories of outcome variables between groups over time. The models were tested for the impact of participant characteristics (age, education, household income, marital status, and nerve-sparing surgery) and, in linear mixed models, secondary outcomes as well (physical activity and pelvic floor muscle exercises). This was done by including them in the models consecutively.

### Ethics Approval

The study received ethics approval from the Regional Ethics Committee (reference 2016/484-31; 2017/512-32; and 2018/147-32) in Sweden.

### Informed Consent and Participation

All men provided written informed consent at inclusion and were informed that they could terminate their participation at any time without giving a reason and that this would not affect their health care. No compensation was provided for participation in the study. All data were processed confidentially.

## Results

### Characteristics of Participants at Inclusion and Baseline Analysis

The characteristics of the participants (Table 1) were similar across the groups at inclusion. In brief, participants were aged 48 to 78 (mean 64, SD 6.23) years. The largest proportion of participants (79/165, 47.9%) was treated with bilateral nerve-sparing surgery, whereas 32.1% (53/165) were treated with unilateral surgery and 20% (33/165) without nerve-sparing surgery. For the majority (101/165, 61.2%), the hospital stay was 1 day, with a range of up to 19 days. Five men stayed in the hospital for >4 days because of complications (eg, hemorrhage, hernia, or infection).

The intervention and control groups were similar in terms of outcome variables at baseline, that is, urinary continence, sexual function, and physical activity (pelvic floor muscle exercises were not measured; Table 1).

**Table 1:** Characteristics of the participants and baseline data (n=165).

Characteristic		Intervention group (n=83)	Control group (n=82)	*P* value	
Age (years), mean (SD)		64 (6.2)	64 (6.3)	.68^a^	
**Marital status, n (%)**					.82^b^
	Single	22 (27)	23 (28)		
	Married or partner	36 (43)	44 (54)		
**Education, n (%)**					.74^c^
	Primary school (9 years)	10 (12)	11 (13)		
	Upper secondary school	22 (27)	20 (24)		
	Folk high school or vocational school	4 (5)	11 (13)		
	University	22 (27)	24 (29)		
**Household monthly income (SEK^d^), n (%)**					.51^c^
	0 to 14,999	3 (4)	1 (1)		
	15,000 to 29,999	10 (12)	14 (17)		
	30,000 to 44,999	12 (14)	20 (24)		
	≥45,000	33 (40)	32 (39)		
**Nerve-sparing surgery, n (%)**					.82^c^
	No	17 (20)	16 (20)		
	Unilateral	25 (30)	28 (34)		
	Bilateral	41 (49)	38 (46)		
Gleason score, mean (SD)		6.9 (0.5)	7.1 (0.6)	.05^a^	
**Sexual function, n (%)**					.55^c^
	Very poor	8 (10)	8 (10)		
	Poor	14 (17)	6 (7)		
	Moderate	8 (10)	22 (27)		
	Good	21 (25)	20 (24)		
	Very good	6 (7)	8 (10)		
**Urinary continence, n (%)**					.65^c^
	Leakage more than once a day	4 (5)	2 (2)		
	Leakage about once a day	0 (0)	1 (1)		
	Leakage more than once a week	3 (4)	6 (7)		
	Leakage about once a week	3 (4)	5 (6)		
	Seldom or no leakage	48 (58)	52 (63)		
**Physical activity, n (%)**					.35^c^
	Sedentary	3 (4)	1 (1)		
	Some physical activity for at least 4 hours per week	25 (30)	29 (35)		
	Regular moderate physical exercise at least 2 to 3 hours per week	27 (33)	27 (33)		
	Regular hard training and competitive sports	2 (2)	8 (10)		

^a^Independent sample *t* test.

^b^Chi-square test.

^c^Mann-Whitney *U* test.

^d^SEK: Swedish Crown (SEK 1=US $0.12).

### Effects on Primary Outcome Measures: Urinary Continence and Sexual Function

#### Linear Mixed Models for Urinary Continence and Sexual Function

Four linear mixed models were used to determine whether ePATH improved the primary outcomes urinary continence (Table 2) and sexual function (Table 3).

Model 1 provided results for urinary continence (Table 2). No statistically significant differences in the changes over time were found between the intervention and control groups (interaction). Therefore, the interaction term is excluded from the model. Urinary continence did not significantly differ between the intervention and control groups (*P*=.09). There was a statistically significant effect showing increasing levels of urinary continence over time in both the groups (all *P*<.001). Investigations of the impact of participant characteristics (age, education, household income, marital status, and nerve-sparing surgery) and secondary outcomes (physical activity and pelvic floor muscle exercises) were performed by adding these variables, one by one, to model 1. Model 2 included nerve-sparing surgery, as it statistically significantly affected urinary continence in a positive direction (*P*<.001 and *P*=.01).

Sexual function was assessed using the same procedure (Table 3). The interaction term was omitted from the model, as the analysis revealed no statistically significant differences in changes over time between the intervention and control groups. No statistically significant difference in sexual function was observed between the intervention and control groups (*P*=.97), but time had a statistically significant effect and showed increasing levels of sexual function (*P*<.001 and *P*=.002). Investigations of the impact of participant characteristics (age, education, household income, marital status, and nerve-sparing surgery) and secondary outcomes (physical activity and pelvic floor muscle exercises) were then performed by adding these variables, one by one, to model 3. Model 4 included age (*P*=.01) and nerve-sparing surgery (*P*<.001 and *P*=.003), as they had significant effects. Younger age and bilateral nerve-sparing surgery affected sexual function positively.

**Table 2:** Results from 2 linear mixed model analyses on urinary continence (Expanded Prostate Cancer Index Composite) with fixed effects.

Variable	Model 1^a^		Model 2^a,b^	
	β (95% CI)	*P* value	β (95% CI)	*P* value
Intercept	73.62 (68.33 to 78.92)	<.001	80.75 (74.73 to 86.77)	<.001
Intervention group^c^	−5.60 (−12.15 to 0.96)	.09	−5.82 (−11.99 to 0.35)	.06
1 month^d^	−34.36 (−38.25 to −30.48)	<.001	−34.36 (−38.25 to −30.48)	<.001
3 months^d^	−21.23 (−24.49 to −17.98)	<.001	−21.23 (−24.49 to −17.98)	<.001
6 months^d^	−7.60 (−10.53 to −4.66)	<.001	−7.60 (−10.53 to −4.66)	<.001
No nerve-sparing surgery	N/A^e^	N/A	−18.78 (−26.99 to −10.57)	<.001
Unilateral nerve-sparing surgery	N/A	N/A	−10.00 (−17.03 to 2.99)	.01

^a^The characteristics of the participants (age, education, household income, marital status, and nerve-sparing surgery) and secondary outcomes were investigated as potential confounders and models controlled for interactions between time and group.

^b^Including nerve-sparing surgery; reference=bilateral nerve-sparing surgery.

^c^Reference=control group.

^d^Reference=12 months postoperatively.

^e^N/A: not applicable (not included in model 1).

**Table 3:** Results from 2 linear mixed model analyses on sexual function (Expanded Prostate Cancer Index Composite) with fixed effects.

Variable	Model 3^a^			Model 4^a,b^	
	β (95% CI)	*P* value	β (95% CI)		*P* value
Intercept	30.32 (24.86 to 35.79)	<.001	88.51 (53.47 to 123.56)		<.001
Intervention group^c^	−.12 (−7.05 to 6.81)	.97	−1.24 (−7.49 to 5.02)		.70
1 month^d^	−9.84 (−13.71 to −5.96)	<.001	−9.84 (−13.71 to −5.96)		<.001
3 months^d^	−5.74 (−9.30 to −2.19)	.002	−5.74 (−9.30 to −2.19)		.002
6 months^d^	−1.37 (−4.15 to −1.42)	.34	−1.37 (−4.15 to −1.42)		.34
Age	N/A^e^	N/A	−.78 (−1.32 to −.24)		.01
No nerve-sparing surgery	N/A	N/A	−18.20 (−26.78 to −9.63)		<.001
Unilateral nerve-sparing surgery	N/A	N/A	−10.85 (−18.03 to −3.66)		.003

^a^The characteristics of the participants (age, education, household income, marital status, and nerve-sparing surgery) and secondary outcomes were investigated as potential confounders and models controlled for interactions between time and group.

^b^Controlled for age and nerve-sparing surgery; reference=bilateral nerve-sparing surgery.

^c^Reference=control group.

^d^Reference=12 months postoperatively.

^e^N/A: not applicable (not included in model 3).

#### Longitudinal Changes in Urinary Continence and Sexual Function in Comparison With Baseline

**Figure 2:**
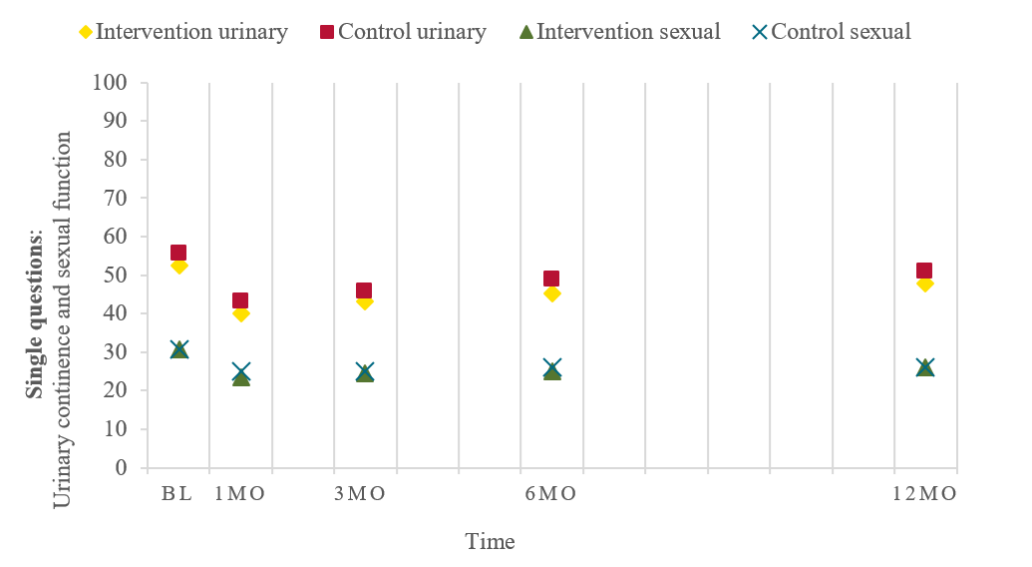
Urinary continence and sexual function (mean) over time in the intervention and control groups. BL: baseline; MO: month.

To illustrate changes over time in urinary continence and sexual function in comparison with baseline, the means of the responses to the single questions (obtained preoperatively) in both groups were plotted (Figure 2), showing decreasing values of urinary continence and sexual function 1 month after surgery, which then increased up to 12 months (in line with linear mixed models; Table 4) without returning to the preoperative levels at baseline.

**Table 4:** Estimated mean values (Expanded Prostate Cancer Index Composite—urinary continence and sexual function) between groups over time (linear mixed model).

Characteristic			1 month, mean (SE; 95% CI)		3 months, mean (SE; 95% CI)		6 months, mean (SE; 95% CI)		12 months, mean (SE; 95% CI)
**Urinary continence**									
	Intervention group	33.66 (2.48; 28.81-38.52)		46.79 (2.66; 41.59-52.00)		60.43 (2.72; 55.10-65.77)		68.03 (2.74; 62.67-73.39)	
	Control group	39.26 (2.46; 34.44-44.08)		52.39 (2.63; 47.24-57.54)		66.03 (2.68; 60.77-71.29)		73.62 (2.70; 68.33-78.92)	
**Sexual function**									
	Intervention group	20.37 (2.63; 15.21-25.53)		24.46 (2.82; 18.94-29.99)		28.84 (2.72; 23.52-34.16)		30.21 (2.79; 24.73-35.68)	
	Control group	20.49 (2.57; 15.45-25.52)		24.58 (2.75; 19.20-29.96)		28.96 (2.72; 23.62-34.30)		30.32 (2.79; 24.86-35.79)	

### Effects on Secondary Outcomes: Physical Activity and Pelvic Floor Muscle Exercises

To study whether ePATH affected the secondary outcomes (physical activity and pelvic floor muscle exercises) over time, ordinal regression analyses were performed (Table 5). No statistically significant interaction effect was found, indicating that there were no differences in the changes over time between the intervention and control groups. Therefore, the interaction term was excluded. ePATH did not show any significant effect on physical activity (*P*=.57), but physical activity increased after 1 month in both groups (model 1). There was no statistically significant difference in pelvic floor muscle exercises between the intervention and control groups (*P*=.15). Pelvic floor muscle exercises decreased over time in both groups (model 2). Participant characteristics (age, education, household income, marital status, and nerve-sparing surgery) were added to the models investigating the effects; no statistically significant impacts were found.

**Table 5:** Results from ordinal regression analyses (generalized estimating equations) on self-care activities: physical activity and pelvic floor muscle exercises.

Variable	Model 1: physical activity^a^		Model 2: pelvic floor muscle exercises^a^	
	Odds ratio (95% CI)	*P* value	Odds ratio (95% CI)	*P* value
Intervention group^b^	1.16 (0.71-1.89)	.57	1.51 (0.86-2.66)	.15
1 month^c^	0.41 (0.23-0.73)	.004	14.45 (9.50-22.00)	<.001
3 months^c^	0.97 (0.62-1.52)	.91	4.15 (2.85-6.03)	<.001
6 months^c^	0.99 (0.69-1.42)	.97	1.20 (1.63-3.35)	<.001

^a^The characteristics of the participants (age, education, household income, marital status, and nerve-sparing surgery) were investigated as potential confounders and models controlled for interactions between time and group.

^b^Reference=control group.

^c^Reference=12 months postoperatively.

### Use

In the intervention group, 64% (53/83) of the participants were defined as users. Use ranged between 1 and 28,214 activities (log-ins or registrations of self-care) over 12 months and declined over time. The median for use was 70 (IQR 6-2330). Nonusers of ePATH accounted for 36% (30/83) of the sample. Of the 53 users, 36 (68%) used ePATH during the first month postoperatively. In total, 32% (17/53) of the participants still used ePATH after 3 months, and the number of users had declined to 21% (11/53) at 6 months. One year after surgery, 11% (6/53) of the men were still using ePATH.

## Discussion

### Principal Findings

The main findings of this randomized controlled trial in cancer rehabilitation were that digital self-care support showed no statistically significant effects on urinary continence or sexual function but increasing levels of urinary continence and sexual function in both the intervention and control groups over time. Previous research shows increasing functioning and decreasing side effects after prostate cancer surgery over time [1], in line with what was shown in both the intervention and control groups in our study. However, estimates and definitions of urinary continence vary among studies, making evaluations difficult to assess and compare. The mechanisms for increasing urinary continence are multifactorial, depending on, for example, surgical technique, anatomical conditions, and self-care technique [3], which may not have been fully captured in this study (ie, body weight or BMI, measurements of obesity, were not included in the study). Individual variations and preferences might impact self-care results, although adherence is crucial to achieve any effect [14]. Therefore, recommendations and support must be customized based on these aspects. This study highlights the need to explore self-care interventions that improve well-being and minimize postoperative complications and to further investigate when support is needed during rehabilitation. The declining use of ePATH over time indicates that this type of support may be appropriate when new behaviors are to be learned and less useful in the long term. It is possible that the need for support in ePATH declined in parallel with the side effects. Previous research shows that patients progress through phases in self-care management, which correspond to differing support needs. When a patient finds routines for self-care and functions gradually return, the patient’s need for support changes [15]. Although the study did not yield the anticipated outcomes, it contributes to the current body of knowledge by emphasizing the significance of providing continuous support to men during their postcancer recovery phase [50]. The maintenance of self-care practices within the home environment is particularly crucial, considering the growing population of patients undergoing rehabilitation owing to improved survival rates [1].

No differences were observed in the effectiveness of ePATH in relation to sexual function between the intervention and control groups. The support delivered through ePATH regarding sexual function entailed physical activity, pelvic floor muscle exercises, and supplementary information regarding sexual rehabilitation. However, sexual rehabilitation is a multifaceted matter that may require multiple different approaches over a longer period and extend to any partner [51-54]. Support and follow-up during the first year after surgery might not encompass all relevant aspects (eg, psychological impact, relationship status, and possibility of using pharmacological agents). It should be noted that our results were not controlled for pharmacological agents in penile rehabilitation. Future research should prioritize the development and assessment of comprehensive web-based sexual rehabilitation support that is adaptable to various contexts.

Self-care regarding physical activity and pelvic floor muscle exercises was not affected by ePATH; however, physical activity increased postoperatively. This is in line with previous research [55], which shows a gradual return to physical activity after surgery. Exercise is increasingly seen as being significant in prostate cancer rehabilitation as a strategy to enhance sexual function, improve feelings of masculinity, and reduce the distress that men experience after prostate cancer [51]. However, urinary incontinence after radical prostatectomy may hinder physical activities [15]. As urinary continence increased, physical activity also increased, thus supporting this notion. Therefore, support from health care might be needed to emphasize the importance of modified physical activity in the early phases of rehabilitation. In our study, pelvic floor muscle exercise decreased over time. Pelvic floor muscle exercises should preferably be guided by a therapist [17,56] or biofeedback [57] to ensure correct technique and achieve an effect on urinary continence. However, such guidance is not standard [7]. Our results highlight the need for additional support for patients to stay adherent to recommendations on pelvic floor muscle exercises in the long term, and a digital app with reminders was not sufficient. Expanded gamification elements and automatic responses incorporated into digital self-care support may encourage adherence to recommendations [58]. For gamification to be relevant for users, established theories on user experiences and the psychological effects of gaming mechanics would need to be applied in the design of eHealth solutions [59].

Although several studies provide evidence of improved health outcomes when using eHealth services [60,61], the evidence remains inconclusive [62]. Reinhardt et al [63] showed that both user-related barriers and intervention-related barriers were common when eHealth tools were used, and digital support does not suit everyone [64,65]. Although there are challenges in evaluating technologically complex interventions in health care, knowledge can be drawn from programs where predicted outcomes do not occur [66]. We explored user needs [16] and based the digital self-care support on theory and evidence [38]. Our pilot study showed promising usability and feasibility [27]. Although people living in the aftermath of cancer treatment often search for information and accessible and effective support tools on the web [26], 36% (30/83) of the participants in the intervention group did not use ePATH. The study design limits the possibility of drawing conclusions regarding nonusers. Qualitative research in the same patient group reported that some men do not feel any need for support [15] or did not have the energy to engage in digital self-care support, as their overall health was poor [27]. Changing behavior is generally difficult, and managing a cancer diagnosis adds another layer to this. There is evidence that different user characteristics are associated with different use patterns; for example, patients with low levels of social support and a high illness burden may find eHealth tools particularly useful [67,68]. In our study, it was unclear whether differences in use could be attributed to comorbidity, symptom distress, support from cancer nurse specialists, need for support, or ePATH per se. Further investigations should explore when, why, and for whom digital self-care support is useful and for whom it is less suitable, so as to adapt digital support to different patient groups.

Although digitization is a top priority in the global health and development sectors, the implementation of innovative interventions and new practices in standard care shows slow progress [69]. Before implementation, a thorough investigation of cost efficiency should be performed to evaluate the clinical relevance of the intervention for patients and in the organization. However, digital interventions have proven to be cost-effective, but further focus is needed on their implementation [70]. The success of an intervention relies on the complex interplay between barriers and enablers, which can determine its effectiveness [71]. Barriers to implementation can take various forms, including poor contextual alignment and systemic factors such as organizational culture and ineffective communication. However, the readiness for change among staff members is particularly crucial, as it affects their willingness and preparedness to adopt behavioral changes and adapt to new care processes [72]. ePATH was added as a complement to standard care to mimic the clinical reality in which implementation could be possible and strengthen the external validity [29]. However, the nurses’ overall workload might have affected their likelihood of engaging in the ePATH intervention. For successful integration of digital interventions, technology needs to be aligned with the organization structure and with the daily processes and user goals [73]. Thus, nurses’ limited time to follow-up on rehabilitation activities might have impacted the outcomes and use. The effectiveness of digital behavioral change interventions in cancer rehabilitation is also dependent on users’ digital and health literacy, attitudes, and engagement and how well the patient needs and contents of the intervention align [36].

### Strengths and Limitations

Multiple strengths and limitations must be considered when evaluating a complex intervention in a randomized controlled trial, in particular external validity and applicability. External factors could have affected the outcomes, as not all influencing factors can be controlled for in a complex trial. The outcome measures used were validated patient-reported outcome measures, which have shown good reliability and validity [41-43]. Using objective measures for physical activity or incontinence (eg, accelerometers or weighing pads) could have increased reliability, but this would risk adding to the burden on participants. As ePATH necessitated an internet connection and a secure mobile log-in facility, the study did not reach certain populations [64,65]. However, eHealth trials that require internet connection, particularly self-help applications, generally have high dropout rates [74]. We applied broad inclusion criteria to reach more participants and did not assess eHealth literacy, technological acceptance, or attitude toward technology [68], which might have had an impact on heterogeneity with regard to patient attrition. The possibility exists that the men who chose not to participate differed from those who were enrolled in the study, potentially impacting the generalizability of the findings. Therefore, it is important to consider the potential impact of self-selection bias when interpreting the results and drawing conclusions about their generalizability to a broader population.

The use and functionality of the application must also be considered. Reminders sent to participants from the research group may have increased use and adherence to the intervention. However, because the study design focused on investigating the intervention in real-world clinical settings rather than ideal circumstances, it was not possible to make such interferences. Consequently, the results of this study are valuable for enhancing our understanding of the nuances and complexities of real-world scenarios, thereby improving the relevance and applicability of research findings [29]. A process evaluation of contextual factors in parallel with the study period would have uncovered barriers and potential improvements of the intervention that may have been useful for implementation in routine care [31,69-72]. However, a lesson learned is that there is probably a need for specific efforts to change work routines and enhance patients’ adherence to prostate cancer rehabilitation.

The planned sample size of 228 randomly assigned participants would have provided at least 80% power to show differences between the groups in this modified intention-to-treat analysis [34]. As the recruitment of participants was slower than expected, enrollment ended at a sample size of 170 for the timeline of the study to be reasonable. The study participants were followed up according to the protocol. Two domains of the EPIC were not included in the analysis despite the power calculations being based on all 4 domains.

A strength is that the repeated measures with 4 assessment points and the use of linear mixed models enabled the inclusion of participants with at least 1 assessment point, which improved the representativeness of the sample [49]. The imputation of missing values should be considered. However, to enhance validity, a widely accepted imputation method was used, and a sensitivity analysis was conducted. To ensure the validity of the results, patients without outcome measures were excluded from imputations (modified intention-to-treat analysis) [45,46]. Imputation and analysis models that are compatible have been shown to result in consistent estimates of both regression parameters and variance components [75]. It is important to consider the dynamic nature of the repeated variables and control effects in the ordinal regression models when interpreting the results.

### Conclusions

Digital solutions have been launched as cancer rehabilitation support in clinical practice, often without sufficient evidence of their benefits. This study adds to the body of knowledge by conducting an effectiveness test of digital self-care support as an adjunct to standard care in real-world conditions. Although this study did not reveal any benefits of rehabilitation after prostatectomy, it provides evidence that comprehensively reflects how this support may function in its clinical context. To optimize support for prostate cancer rehabilitation, further efforts for continued motivation and the use of digital support need to be considered. Future research should focus on user requirements and timing of support in the population with prostate cancer as well as structural preconditions for implementing effective digital support in existing work processes.

## Appendix

Multimedia Appendix 1 CONSORT-eHEALTH checklist (V 1.6.1).

## Abbreviations

CONSORT-EHEALTH: Consolidated Standards of Reporting Trials of Electronic and Mobile Health Applications and Online Telehealth
ePATH: electronic Patient Activation in Treatment at Home
EPIC: Expanded Prostate Cancer Index Composite

## Data Availability

The data sets generated and analyzed during this study are not publicly available because of ethics approval concerning the handling of the gathered data. However, they are available from the corresponding author upon reasonable request.
