# Supplementary material for: Effects of Web-Based and Mobile Self-Care Support in Addition to Standard Care in Patients After Radical Prostatectomy: Randomized Controlled Trial
Source: JMIR Cancer. 2023 Sep 6;9:e44320. doi: 10.2196/44320 (PMC10512115; doi:10.2196/44320)
Supplement: Multimedia Appendix 1 [file cancer_v9i1e44320_app1.pdf]

CONSORT-EHEALTH (V 1.6.1) - Submission/Publication Form

The CONSORT-EHEALTH checklist is intended for authors of randomized trials evaluating web-based and Internet-based applications/interventions, including mobile interventions, electronic games (incl multiplayer games), social media, certain telehealth applications, and other interactive and/or networked electronic applications. Some of the items (e.g. all subitems under item 5 - description of the intervention) may also be applicable for other study designs.

The goal of the CONSORT EHEALTH checklist and guideline is to be
a) a guide for reporting for authors of RCTs,
b) to form a basis for appraisal of an ehealth trial (in terms of validity)

CONSORT-EHEALTH items/subitems are MANDATORY reporting items for studies published in the Journal of Medical Internet Research and other journals / scientific societies endorsing the checklist.

Items numbered 1., 2., 3., 4a., 4b etc are original CONSORT or CONSORT-NPT (non-pharmacologic treatment) items.
Items with Roman numerals (i., ii, iii, iv etc.) are CONSORT-EHEALTH extensions/clarifications.

As the CONSORT-EHEALTH checklist is still considered in a formative stage, we would ask that you also RATE ON A SCALE OF 1-5 how important/useful you feel each item is FOR THE PURPOSE OF THE CHECKLIST and reporting guideline (optional).

Mandatory reporting items are marked with a red *.
In the textboxes, either copy & paste the relevant sections from your manuscript into this form - please include any quotes from your manuscript in QUOTATION MARKS,
or answer directly by providing additional information not in the manuscript, or elaborating on why the item was not relevant for this study.

YOUR ANSWERS WILL BE PUBLISHED AS A SUPPLEMENTARY FILE TO YOUR PUBLICATION IN JMIR AND ARE CONSIDERED PART OF YOUR PUBLICATION (IF ACCEPTED).
Please fill in these questions diligently. Information will not be copyedited, so please use proper spelling and grammar, use correct capitalization, and avoid abbreviations.

DO NOT FORGET TO SAVE AS PDF _AND_ CLICK THE SUBMIT BUTTON SO YOUR ANSWERS ARE IN OUR DATABASE !!!

Citation Suggestion (if you append the pdf as Appendix we suggest to cite this paper in the caption):
Eysenbach G, CONSORT-EHEALTH Group
[illegible]
J Med Internet Res 2011;13(4):e126

Ditt svar är för stort. Testa att förkorta en del av dina svar.

URL: http://www.jmir.org/2011/4/e126/
doi: 10.2196/jmir.1923
PMID: 22209829

camillawennerberg@gmail.com (Delas inte) Byt konto

Utkastet har sparats

*Obligatorisk

Your name *

First Last

Camilla Wennerberg

Primary Affiliation (short), City, Country *

University of Toronto, Toronto, Canada

Linnaeus University, Kalmar, Sweden

Your e-mail address *

abc@gmail.com

camilla.wennerberg@lnu.se

Title of your manuscript *

Provide the (draft) title of your manuscript.

Effects of a web-based and mobile self-care support in addition standard care, in patients after radical prostatectomy: A randomized controlled trial

Ditt svar är för stort. Testa att förkorta en del av dina svar.

Name of your App/Software/Intervention *

If there is a short and a long/alternate name, write the short name first and add the long name in brackets.

ePATH (electronic Patient Activation in Treatm

Evaluated Version (if any)

e.g. "V1", "Release 2017-03-01", "Version 2.0.27913"

Ditt svar

Language(s) *

What language is the intervention/app in? If multiple languages are available, separate by comma (e.g. "English, French")

Swedish

URL of your Intervention Website or App

e.g. a direct link to the mobile app on app in appstore (itunes, Google Play), or URL of the website. If the intervention is a DVD or hardware, you can also link to an Amazon page.

https://epath.se/ (no longer in use)

URL of an image/screenshot (optional)

https://epath.se/ (no longer in use)

Ditt svar är för stort. Testa att förkorta en del av dina svar.

Accessibility *

Can an enduser access the intervention presently?

- ○ access is free and open
- ○ access only for special usergroups, not open
- ○ access is open to everyone, but requires payment/subscription/in-app purchases
- ◉ app/intervention no longer accessible
- ○ Övrigt:

Primary Medical Indication/Disease/Condition *

e.g. "Stress", "Diabetes", or define the target group in brackets after the condition, e.g. "Autism (Parents of children with)", "Alzheimers (Informal Caregivers of)"

Prostate cancer (radical prostatectomy)

Primary Outcomes measured in trial *

comma-separated list of primary outcomes reported in the trial

Urinary Continence, Sexual Function

Secondary/other outcomes

Are there any other outcomes the intervention is expected to affect?

Adherence to self-care, pelvic floor muscle exercises and physical activity

Ditt svar är för stort. Testa att förkorta en del av dina svar.

Recommended "Dose" *

What do the instructions for users say on how often the app should be used?

- ○ Approximately Daily
- ○ Approximately Weekly
- ○ Approximately Monthly
- ○ Approximately Yearly
- ◉ "as needed"
- ○ Övrigt:

Approx. Percentage of Users (starters) still using the app as recommended after 3 months *

- ○ unknown / not evaluated
- ○ 0-10%
- ○ 11-20%
- ○ 21-30%
- ◉ 31-40%
- ○ 41-50%
- ○ 51-60%
- ○ 61-70%
- ○ 71%-80%
- ○ 81-90%
- ○ 91-100%
- ○ Övrigt:

Ditt svar är för stort. Testa att förkorta en del av dina svar.

Overall, was the app/intervention effective? *

- ○ yes: all primary outcomes were significantly better in intervention group vs control
- ○ partly: SOME primary outcomes were significantly better in intervention group vs control
- ◉ no statistically significant difference between control and intervention
- ○ potentially harmful: control was significantly better than intervention in one or more outcomes
- ○ inconclusive: more research is needed
- ○ Övrigt:

Article Preparation Status/Stage *

At which stage in your article preparation are you currently (at the time you fill in this form)

- ○ not submitted yet - in early draft status
- ◉ not submitted yet - in late draft status, just before submission
- ○ submitted to a journal but not reviewed yet
- ○ submitted to a journal and after receiving initial reviewer comments
- ○ submitted to a journal and accepted, but not published yet
- ○ published
- ○ Övrigt:

Ditt svar är för stort. Testa att förkorta en del av dina svar.

Journal *

If you already know where you will submit this paper (or if it is already submitted), please provide the journal name (if it is not JMIR, provide the journal name under "other")

- [ ] not submitted yet / unclear where I will submit this
- [x] Journal of Medical Internet Research (JMIR)
- [ ] JMIR mHealth and UHealth
- [ ] JMIR Serious Games
- [ ] JMIR Mental Health
- [ ] JMIR Public Health
- [ ] JMIR Formative Research
- [ ] Other JMIR sister journal
- [ ] Övrigt:

Is this a full powered effectiveness trial or a pilot/feasibility trial? *

- [ ] Pilot/feasibility
- [x] Fully powered

Manuscript tracking number *

If this is a JMIR submission, please provide the manuscript tracking number under "other" (The ms tracking number can be found in the submission acknowledgement email, or when you login as author in JMIR. If the paper is already published in JMIR, then the ms tracking number is the four-digit number at the end of the DOI, to be found at the bottom of each published article in JMIR)

- [x] no ms number (yet) / not (yet) submitted to / published in JMIR
- [ ] Övrigt:

Ditt svar är för stort. Testa att förkorta en del av dina svar.

TITLE AND ABSTRACT

1a) TITLE: Identification as a randomized trial in the title

1a) Does your paper address CONSORT item 1a? *

I.e does the title contain the phrase "Randomized Controlled Trial"? (if not, explain the reason under "other")

- (x) yes
- ( ) Övrigt:

1a-i) Identify the mode of delivery in the title

Identify the mode of delivery. Preferably use “web-based” and/or “mobile” and/or “electronic game” in the title. Avoid ambiguous terms like “online”, “virtual”, “interactive”. Use “Internet-based” only if Intervention includes non-web-based Internet components (e.g. email), use “computer-based” or “electronic” only if offline products are used. Use “virtual” only in the context of “virtual reality” (3-D worlds). Use “online” only in the context of “online support groups”. Complement or substitute product names with broader terms for the class of products (such as “mobile” or “smart phone” instead of “iphone”), especially if the application runs on different platforms.

subitem not at all important

- ( ) 1
- ( ) 2
- (x) 3
- ( ) 4
- ( ) 5

essential

Rensa markering

Ditt svar är för stort. Testa att förkorta en del av dina svar.

Does your paper address subitem 1a-i? *

Copy and paste relevant sections from manuscript title (include quotes in quotation marks "like this" to indicate direct quotes from your manuscript), or elaborate on this item by providing additional information not in the ms, or briefly explain why the item is not applicable/relevant for your study

"web-based and mobile self-care support"

1a-ii) Non-web-based components or important co-interventions in title

Mention non-web-based components or important co-interventions in title, if any (e.g., “with telephone support”).

subitem not at all important

1 ○

2 ○

3 ◉

4 ○

5 ○

essential

Rensa markering

Does your paper address subitem 1a-ii?

Copy and paste relevant sections from manuscript title (include quotes in quotation marks "like this" to indicate direct quotes from your manuscript), or elaborate on this item by providing additional information not in the ms, or briefly explain why the item is not applicable/relevant for your study

Does not include non-web-based or co-interventions

Ditt svar är för stort. Testa att förkorta en del av dina svar.

1a-iii) Primary condition or target group in the title

Mention primary condition or target group in the title, if any (e.g., “for children with Type I Diabetes”) Example: A Web-based and Mobile Intervention with Telephone Support for Children with Type I Diabetes: Randomized Controlled Trial

subitem not at all important

- 1 ○
- 2 ○
- 3 ○
- 4 ○
- 5 ◉

essential

Rensa markering

Does your paper address subitem 1a-iii? *

Copy and paste relevant sections from manuscript title (include quotes in quotation marks "like this" to indicate direct quotes from your manuscript), or elaborate on this item by providing additional information not in the ms, or briefly explain why the item is not applicable/relevant for your study

"in patients after radical prostatectomy"

1b) ABSTRACT: Structured summary of trial design, methods, results, and conclusions

NPT extension: Description of experimental treatment, comparator, care providers, centers, and blinding status.

Ditt svar är för stort. Testa att förkorta en del av dina svar.

1b-i) Key features/functionalities/components of the intervention and comparator in the METHODS section of the ABSTRACT

Mention key features/functionalities/components of the intervention and comparator in the abstract. If possible, also mention theories and principles used for designing the site. Keep in mind the needs of systematic reviewers and indexers by including important synonyms. (Note: Only report in the abstract what the main paper is reporting. If this information is missing from the main body of text, consider adding it)

subitem not at all important

1 ○

2 ○

3 ○

4 ◉

5 ○

essential

Rensa markering

Does your paper address subitem 1b-i? *

Copy and paste relevant sections from the manuscript abstract (include quotes in quotation marks "like this" to indicate direct quotes from your manuscript), or elaborate on this item by providing additional information not in the ms, or briefly explain why the item is not applicable/relevant for your study

"...longitudinal effects of the additional digital self-care support (ePATH) compared with those of standard care alone. ePATH was designed based on the self-determination theory to strengthen patient's activation in self-care through nurse-assisted individualized modules."

Ditt svar är för stort. Testa att förkorta en del av dina svar.

1b-ii) Level of human involvement in the METHODS section of the ABSTRACT

Clarify the level of human involvement in the abstract, e.g., use phrases like “fully automated” vs. “therapist/nurse/care provider/physician-assisted” (mention number and expertise of providers involved, if any). (Note: Only report in the abstract what the main paper is reporting. If this information is missing from the main body of text, consider adding it)

subitem not at all important

1 ○

2 ○

3 ◉

4 ○

5 ○

essential

Rensa markering

Does your paper address subitem 1b-ii?

Copy and paste relevant sections from the manuscript abstract (include quotes in quotation marks "like this" to indicate direct quotes from your manuscript), or elaborate on this item by providing additional information not in the ms, or briefly explain why the item is not applicable/relevant for your study

"nurse-assisted individualized modules"

Ditt svar är för stort. Testa att förkorta en del av dina svar.

1b-iii) Open vs. closed, web-based (self-assessment) vs. face-to-face assessments in the METHODS section of the ABSTRACT

Mention how participants were recruited (online vs. offline), e.g., from an open access website or from a clinic or a closed online user group (closed usergroup trial), and clarify if this was a purely web-based trial, or there were face-to-face components (as part of the intervention or for assessment). Clearly say if outcomes were self-assessed through questionnaires (as common in web-based trials). Note: In traditional offline trials, an open trial (open-label trial) is a type of clinical trial in which both the researchers and participants know which treatment is being administered. To avoid confusion, use "blinded" or "unblinded" to indicated the level of blinding instead of "open", as "open" in web-based trials usually refers to "open access" (i.e. participants can self-enrol). (Note: Only report in the abstract what the main paper is reporting. If this information is missing from the main body of text, consider adding it)

subitem not at all important

1 ○

2 ○

3 ◉

4 ○

5 ○

essential

Rensa markering

Does your paper address subitem 1b-iii?

Copy and paste relevant sections from the manuscript abstract (include quotes in quotation marks "like this" to indicate direct quotes from your manuscript), or elaborate on this item by providing additional information not in the ms, or briefly explain why the item is not applicable/relevant for your study

"Men planned for radical prostatectomy at three county hospitals in southern Sweden were included offline and randomly assigned to the intervention or control group. The effects of ePATH were evaluated for one year after surgery with self-assessed questionnaires."

Ditt svar är för stort. Testa att förkorta en del av dina svar.

1b-iv) RESULTS section in abstract must contain use data

Report number of participants enrolled/assessed in each group, the use/uptake of the intervention (e.g., attrition/adherence metrics, use over time, number of logins etc.), in addition to primary/secondary outcomes. (Note: Only report in the abstract what the main paper is reporting. If this information is missing from the main body of text, consider adding it)

subitem not at all important

1 ○

2 ○

3 ◉

4 ○

5 ○

essential

Rensa markering

Does your paper address subitem 1b-iv?

Copy and paste relevant sections from the manuscript abstract (include quotes in quotation marks "like this" to indicate direct quotes from your manuscript), or elaborate on this item by providing additional information not in the ms, or briefly explain why the item is not applicable/relevant for your study

"The study included 170 men (85 in each group)...Of the intervention group 64% (53/83) used ePATH, but usage declined over time."

Ditt svar är för stort. Testa att förkorta en del av dina svar.

1b-v) CONCLUSIONS/DISCUSSION in abstract for negative trials

Conclusions/Discussions in abstract for negative trials: Discuss the primary outcome - if the trial is negative (primary outcome not changed), and the intervention was not used, discuss whether negative results are attributable to lack of uptake and discuss reasons. (Note: Only report in the abstract what the main paper is reporting. If this information is missing from the main body of text, consider adding it)

subitem not at all important

- 1 ○
- 2 ○
- 3 ◉
- 4 ○
- 5 ○

essential

Rensa markering

Does your paper address subitem 1b-v?

Copy and paste relevant sections from the manuscript abstract (include quotes in quotation marks "like this" to indicate direct quotes from your manuscript), or elaborate on this item by providing additional information not in the ms, or briefly explain why the item is not applicable/relevant for your study

"To complement standard rehabilitation, digital self-care support needs to be adapted to the context and individual preferences for usage and effect."

INTRODUCTION

2a) In INTRODUCTION: Scientific background and explanation of rationale

Ditt svar är för stort. Testa att förkorta en del av dina svar.

2a-i) Problem and the type of system/solution

Describe the problem and the type of system/solution that is object of the study: intended as stand-alone intervention vs. incorporated in broader health care program? Intended for a particular patient population? Goals of the intervention, e.g., being more cost-effective to other interventions, replace or complement other solutions? (Note: Details about the intervention are provided in “Methods” under 5)

subitem not at all important

1 ○

2 ○

3 ○

4 ◉

5 ○

essential

Rensa markering

Does your paper address subitem 2a-i? *

Copy and paste relevant sections from the manuscript (include quotes in quotation marks "like this" to indicate direct quotes from your manuscript), or elaborate on this item by providing additional information not in the ms, or briefly explain why the item is not applicable/relevant for your study

"Goals of the study is to test the effectiveness of a digital self-care support as a complement to standard care - to be integrated in existing electronic channels"

Ditt svar är för stort. Testa att förkorta en del av dina svar.

2a-ii) Scientific background, rationale: What is known about the (type of) system

Scientific background, rationale: What is known about the (type of) system that is the object of the study (be sure to discuss the use of similar systems for other conditions/diagnoses, if appropiate), motivation for the study, i.e. what are the reasons for and what is the context for this specific study, from which stakeholder viewpoint is the study performed, potential impact of findings [2]. Briefly justify the choice of the comparator.

subitem not at all important

1 ○

2 ○

3 ◉

4 ○

5 ○

essential

Rensa markering

Does your paper address subitem 2a-ii? *

Copy and paste relevant sections from the manuscript (include quotes in quotation marks "like this" to indicate direct quotes from your manuscript), or elaborate on this item by providing additional information not in the ms, or briefly explain why the item is not applicable/relevant for your study

"Although some intervention show improved symptom burdens in prostate cancer patients in the first months after surgery, the long-term effects have been only sparsely evaluated in randomized controlled trials."

2b) In INTRODUCTION: Specific objectives or hypotheses

Ditt svar är för stort. Testa att förkorta en del av dina svar.

Does your paper address CONSORT subitem 2b? *

Copy and paste relevant sections from the manuscript (include quotes in quotation marks "like this" to indicate direct quotes from your manuscript), or elaborate on this item by providing additional information not in the ms, or briefly explain why the item is not applicable/relevant for your study

"In this randomized controlled trial, we evaluated whether ePATH (in addition to standard care) improved patient rehabilitation outcomes among men who had undergone radical prostatectomy, as compared with standard care alone, over a 1-year period."

METHODS

3a) Description of trial design (such as parallel, factorial) including allocation ratio

Does your paper address CONSORT subitem 3a? *

Copy and paste relevant sections from the manuscript (include quotes in quotation marks "like this" to indicate direct quotes from your manuscript), or elaborate on this item by providing additional information not in the ms, or briefly explain why the item is not applicable/relevant for your study

"This multicenter block-randomized controlled trial design with two study arms had a longitudinal study design with follow-up measures at 1, 3, 6- and 12-months post-surgery."

3b) Important changes to methods after trial commencement (such as eligibility criteria), with reasons

Does your paper address CONSORT subitem 3b? *

Copy and paste relevant sections from the manuscript (include quotes in quotation marks "like this" to indicate direct quotes from your manuscript), or elaborate on this item by providing additional information not in the ms, or briefly explain why the item is not applicable/relevant for your study

No important changes made

Ditt svar är för stort. Testa att förkorta en del av dina svar.

3b-i) Bug fixes, Downtimes, Content Changes

Bug fixes, Downtimes, Content Changes: ehealth systems are often dynamic systems. A description of changes to methods therefore also includes important changes made on the intervention or comparator during the trial (e.g., major bug fixes or changes in the functionality or content) (5-iii) and other “unexpected events” that may have influenced study design such as staff changes, system failures/downtimes, etc. [2].

subitem not at all important

1 ○

2 ○

3 ○

4 ◉

5 ○

essential

Rensa markering

Does your paper address subitem 3b-i?

Copy and paste relevant sections from the manuscript (include quotes in quotation marks "like this" to indicate direct quotes from your manuscript), or elaborate on this item by providing additional information not in the ms, or briefly explain why the item is not applicable/relevant for your study

"Minor bugs in the system were fixed during the evaluation process, but no changes to the content were made."

4a) Eligibility criteria for participants

Ditt svar är för stort. Testa att förkorta en del av dina svar.

Does your paper address CONSORT subitem 4a? *

Copy and paste relevant sections from the manuscript (include quotes in quotation marks "like this" to indicate direct quotes from your manuscript), or elaborate on this item by providing additional information not in the ms, or briefly explain why the item is not applicable/relevant for your study

Described under "Recruitment"

4a-i) Computer / Internet literacy

Computer / Internet literacy is often an implicit "de facto" eligibility criterion - this should be explicitly clarified.

subitem not at all important

- 1 ○
- 2 ○
- 3 ○
- 4 ●
- 5 ○

essential

Rensa markering

Does your paper address subitem 4a-i?

Copy and paste relevant sections from the manuscript (include quotes in quotation marks "like this" to indicate direct quotes from your manuscript), or elaborate on this item by providing additional information not in the ms, or briefly explain why the item is not applicable/relevant for your study

"being computer literate (assessed by each man himself)"

Ditt svar är för stort. Testa att förkorta en del av dina svar.

4a-ii) Open vs. closed, web-based vs. face-to-face assessments:

Open vs. closed, web-based vs. face-to-face assessments: Mention how participants were recruited (online vs. offline), e.g., from an open access website or from a clinic, and clarify if this was a purely web-based trial, or there were face-to-face components (as part of the intervention or for assessment), i.e., to what degree got the study team to know the participant. In online-only trials, clarify if participants were quasi-anonymous and whether having multiple identities was possible or whether technical or logistical measures (e.g., cookies, email confirmation, phone calls) were used to detect/prevent these.

subitem not at all important

1 ○

2 ○

3 ○

4 ◉

5 ○

essential

Rensa markering

Does your paper address subitem 4a-ii? *

Copy and paste relevant sections from the manuscript (include quotes in quotation marks "like this" to indicate direct quotes from your manuscript), or elaborate on this item by providing additional information not in the ms, or briefly explain why the item is not applicable/relevant for your study

Men were included offline by there cancer nurse specialist in clinic. No face-to-face components after inclusion.

Ditt svar är för stort. Testa att förkorta en del av dina svar.

4a-iii) Information giving during recruitment

Information given during recruitment. Specify how participants were briefed for recruitment and in the informed consent procedures (e.g., publish the informed consent documentation as appendix, see also item X26), as this information may have an effect on user self-selection, user expectation and may also bias results.

subitem not at all important

1 ○

2 ○

3 ○

4 ○

5 ◉

essential

Rensa markering

Does your paper address subitem 4a-iii?

Copy and paste relevant sections from the manuscript (include quotes in quotation marks "like this" to indicate direct quotes from your manuscript), or elaborate on this item by providing additional information not in the ms, or briefly explain why the item is not applicable/relevant for your study

"All men planned for radical prostatectomy at the three hospitals got preoperative written information about the study and a manual for ePATH from their cancer nurse specialist, in conjunction with the treatment decision. Roughly a week after receiving written information, the men were asked verbally by the cancer nurse specialist about participation; if they chose to participate, a signed consent form was sent by post to the researchers."

4b) Settings and locations where the data were collected

Ditt svar är för stort. Testa att förkorta en del av dina svar.

Does your paper address CONSORT subitem 4b? *

Copy and paste relevant sections from the manuscript (include quotes in quotation marks "like this" to indicate direct quotes from your manuscript), or elaborate on this item by providing additional information not in the ms, or briefly explain why the item is not applicable/relevant for your study

Described under "Participants and Setting" in manuscript.

4b-i) Report if outcomes were (self-)assessed through online questionnaires

Clearly report if outcomes were (self-)assessed through online questionnaires (as common in web-based trials) or otherwise.

subitem not at all important

- 1 ○
- 2 ◉
- 3 ○
- 4 ○
- 5 ○

essential

Rensa markering

Does your paper address subitem 4b-i? *

Copy and paste relevant sections from the manuscript (include quotes in quotation marks "like this" to indicate direct quotes from your manuscript), or elaborate on this item by providing additional information not in the ms, or briefly explain why the item is not applicable/relevant for your study

Outcomes were self-assessed through questionnaires by link in email.

Ditt svar är för stort. Testa att förkorta en del av dina svar.

4b-ii) Report how institutional affiliations are displayed

Report how institutional affiliations are displayed to potential participants [on ehealth media], as affiliations with prestigious hospitals or universities may affect volunteer rates, use, and reactions with regards to an intervention.(Not a required item – describe only if this may bias results)

subitem not at all important

1 ○

2 ○

3 ◉

4 ○

5 ○

essential

Rensa markering

Does your paper address subitem 4b-ii?

Copy and paste relevant sections from the manuscript (include quotes in quotation marks "like this" to indicate direct quotes from your manuscript), or elaborate on this item by providing additional information not in the ms, or briefly explain why the item is not applicable/relevant for your study

Responsible University displayed in web-based and mobile application. Patients were included offline and institutional affiliation had been included in the offline informed consent as well.

5) The interventions for each group with sufficient details to allow replication, including how and when they were actually administered

Ditt svar är för stort. Testa att förkorta en del av dina svar.

5-i) Mention names, credential, affiliations of the developers, sponsors, and owners

Mention names, credential, affiliations of the developers, sponsors, and owners [6] (if authors/evaluators are owners or developer of the software, this needs to be declared in a "Conflict of interest" section or mentioned elsewhere in the manuscript).

subitem not at all important

1 ○

2 ○

3 ○

4 ◉

5 ○

essential

Rensa markering

Does your paper address subitem 5-i?

Copy and paste relevant sections from the manuscript (include quotes in quotation marks "like this" to indicate direct quotes from your manuscript), or elaborate on this item by providing additional information not in the ms, or briefly explain why the item is not applicable/relevant for your study

Described under "Intervention" in manuscript.

Ditt svar är för stort. Testa att förkorta en del av dina svar.

5-ii) Describe the history/development process

Describe the history/development process of the application and previous formative evaluations (e.g., focus groups, usability testing), as these will have an impact on adoption/use rates and help with interpreting results.

subitem not at all important

1 ○

2 ○

3 ○

4 ◉

5 ○

essential

Rensa markering

Does your paper address subitem 5-ii?

Copy and paste relevant sections from the manuscript (include quotes in quotation marks "like this" to indicate direct quotes from your manuscript), or elaborate on this item by providing additional information not in the ms, or briefly explain why the item is not applicable/relevant for your study

Described under "Intervention" in manuscript and in previous publications.

Ditt svar är för stort. Testa att förkorta en del av dina svar.

5-iii) Revisions and updating

Revisions and updating. Clearly mention the date and/or version number of the application/intervention (and comparator, if applicable) evaluated, or describe whether the intervention underwent major changes during the evaluation process, or whether the development and/or content was “frozen” during the trial. Describe dynamic components such as news feeds or changing content which may have an impact on the replicability of the intervention (for unexpected events see item 3b).

subitem not at all important

1 ○

2 ○

3 ○

4 ○

5 ◉

essential

Rensa markering

Does your paper address subitem 5-iii?

Copy and paste relevant sections from the manuscript (include quotes in quotation marks "like this" to indicate direct quotes from your manuscript), or elaborate on this item by providing additional information not in the ms, or briefly explain why the item is not applicable/relevant for your study

"Minor bugs were fixed in the system during the evaluation process, but no changes to the content were made."

Ditt svar är för stort. Testa att förkorta en del av dina svar.

5-iv) Quality assurance methods

Provide information on quality assurance methods to ensure accuracy and quality of information provided [1], if applicable.

subitem not at all important

1 ○

2 ○

3 ◉

4 ○

5 ○

essential

Rensa markering

Does your paper address subitem 5-iv?

Copy and paste relevant sections from the manuscript (include quotes in quotation marks "like this" to indicate direct quotes from your manuscript), or elaborate on this item by providing additional information not in the ms, or briefly explain why the item is not applicable/relevant for your study

Described under "Intervention" in manuscript and in previous publications.

Ditt svar är för stort. Testa att förkorta en del av dina svar.

5-v) Ensure replicability by publishing the source code, and/or providing screenshots/screen-capture video, and/or providing flowcharts of the algorithms used

Ensure replicability by publishing the source code, and/or providing screenshots/screen-capture video, and/or providing flowcharts of the algorithms used. Replicability (i.e., other researchers should in principle be able to replicate the study) is a hallmark of scientific reporting.

subitem not at all important

1 ○

2 ○

3 ◉

4 ○

5 ○

essential

Rensa markering

Does your paper address subitem 5-v?

Copy and paste relevant sections from the manuscript (include quotes in quotation marks "like this" to indicate direct quotes from your manuscript), or elaborate on this item by providing additional information not in the ms, or briefly explain why the item is not applicable/relevant for your study

Screenshots are published alongside with the article. Further information in the previously published articles.

Ditt svar är för stort. Testa att förkorta en del av dina svar.

5-vi) Digital preservation

Digital preservation: Provide the URL of the application, but as the intervention is likely to change or disappear over the course of the years; also make sure the intervention is archived (Internet Archive, webcitation.org, and/or publishing the source code or screenshots/videos alongside the article). As pages behind login screens cannot be archived, consider creating demo pages which are accessible without login.

subitem not at all important

1 ○

2 ○

3 ◉

4 ○

5 ○

essential

Rensa markering

Does your paper address subitem 5-vi?

Copy and paste relevant sections from the manuscript (include quotes in quotation marks "like this" to indicate direct quotes from your manuscript), or elaborate on this item by providing additional information not in the ms, or briefly explain why the item is not applicable/relevant for your study

Screenshots are published alongside with the article. Further information in the previously published articles.

Ditt svar är för stort. Testa att förkorta en del av dina svar.

5-vii) Access

Access: Describe how participants accessed the application, in what setting/context, if they had to pay (or were paid) or not, whether they had to be a member of specific group. If known, describe how participants obtained “access to the platform and Internet” [1]. To ensure access for editors/reviewers/readers, consider to provide a “backdoor” login account or demo mode for reviewers/readers to explore the application (also important for archiving purposes, see vi).

subitem not at all important

1 ○

2 ○

3 ○

4 ◉

5 ○

essential

Rensa markering

Does your paper address subitem 5-vii? *

Copy and paste relevant sections from the manuscript (include quotes in quotation marks "like this" to indicate direct quotes from your manuscript), or elaborate on this item by providing additional information not in the ms, or briefly explain why the item is not applicable/relevant for your study

ePATH was free of charge. "...having a facility for secure mobile login...Those in the intervention group also received a message in ePATH, letting them know when their account was ready to use."

Screenshots are published alongside with the article.

Ditt svar är för stort. Testa att förkorta en del av dina svar.

5-viii) Mode of delivery, features/functionalities/components of the intervention and comparator, and the theoretical framework

Describe mode of delivery, features/functionalities/components of the intervention and comparator, and the theoretical framework [6] used to design them (instructional strategy [1], behaviour change techniques, persuasive features, etc., see e.g., [7, 8] for terminology). This includes an in-depth description of the content (including where it is coming from and who developed it) [1]," whether [and how] it is tailored to individual circumstances and allows users to track their progress and receive feedback" [6]. This also includes a description of communication delivery channels and – if computer-mediated communication is a component – whether communication was synchronous or asynchronous [6]. It also includes information on presentation strategies [1], including page design principles, average amount of text on pages, presence of hyperlinks to other resources, etc. [1].

subitem not at all important

1 ○

2 ○

3 ◉

4 ○

5 ○

essential

Rensa markering

Does your paper address subitem 5-viii? *

Copy and paste relevant sections from the manuscript (include quotes in quotation marks "like this" to indicate direct quotes from your manuscript), or elaborate on this item by providing additional information not in the ms, or briefly explain why the item is not applicable/relevant for your study

Described under "Intervention" in manuscript.

Ditt svar är för stort. Testa att förkorta en del av dina svar.

5-ix) Describe use parameters

Describe use parameters (e.g., intended “doses” and optimal timing for use). Clarify what instructions or recommendations were given to the user, e.g., regarding timing, frequency, heaviness of use, if any, or was the intervention used ad libitum.

subitem not at all important

1 ○

2 ○

3 ◉

4 ○

5 ○

essential

Rensa markering

Does your paper address subitem 5-ix?

Copy and paste relevant sections from the manuscript (include quotes in quotation marks "like this" to indicate direct quotes from your manuscript), or elaborate on this item by providing additional information not in the ms, or briefly explain why the item is not applicable/relevant for your study

"The participants could use and personalize their account to fit individual circumstances and preferences in whatever way they preferred, like times of day or days of the week to perform exercises [33]."

Ditt svar är för stort. Testa att förkorta en del av dina svar.

5-x) Clarify the level of human involvement

Clarify the level of human involvement (care providers or health professionals, also technical assistance) in the e-intervention or as co-intervention (detail number and expertise of professionals involved, if any, as well as “type of assistance offered, the timing and frequency of the support, how it is initiated, and the medium by which the assistance is delivered”. It may be necessary to distinguish between the level of human involvement required for the trial, and the level of human involvement required for a routine application outside of a RCT setting (discuss under item 21 – generalizability).

subitem not at all important

1 ○

2 ○

3 ◉

4 ○

5 ○

essential

Rensa markering

Does your paper address subitem 5-x?

Copy and paste relevant sections from the manuscript (include quotes in quotation marks "like this" to indicate direct quotes from your manuscript), or elaborate on this item by providing additional information not in the ms, or briefly explain why the item is not applicable/relevant for your study

"ePATH also included a function for messaging the cancer nurse specialists for support and guidance, through a secure pathway. The cancer nurse specialists followed up and answered patient messages daily, but did not check patient engagement in ePATH."

Ditt svar är för stort. Testa att förkorta en del av dina svar.

5-xi) Report any prompts/reminders used

Report any prompts/reminders used: Clarify if there were prompts (letters, emails, phone calls, SMS) to use the application, what triggered them, frequency etc. It may be necessary to distinguish between the level of prompts/reminders required for the trial, and the level of prompts/reminders for a routine application outside of a RCT setting (discuss under item 21 – generalizability).

subitem not at all important

1 ○

2 ○

3 ◉

4 ○

5 ○

essential

Rensa markering

Does your paper address subitem 5-xi? *

Copy and paste relevant sections from the manuscript (include quotes in quotation marks "like this" to indicate direct quotes from your manuscript), or elaborate on this item by providing additional information not in the ms, or briefly explain why the item is not applicable/relevant for your study

It was optional for participants in the intervention group to activate notifications (reminders) in the app to perform self-care.

Ditt svar är för stort. Testa att förkorta en del av dina svar.

5-xii) Describe any co-interventions (incl. training/support)

Describe any co-interventions (incl. training/support): Clearly state any interventions that are provided in addition to the targeted eHealth intervention, as ehealth intervention may not be designed as stand-alone intervention. This includes training sessions and support [1]. It may be necessary to distinguish between the level of training required for the trial, and the level of training for a routine application outside of a RCT setting (discuss under item 21 – generalizability.

subitem not at all important

1 ○

2 ○

3 ◉

4 ○

5 ○

essential

Rensa markering

Does your paper address subitem 5-xii? *

Copy and paste relevant sections from the manuscript (include quotes in quotation marks "like this" to indicate direct quotes from your manuscript), or elaborate on this item by providing additional information not in the ms, or briefly explain why the item is not applicable/relevant for your study

"All men planned for radical prostatectomy receive written pamphlets on self-care, as well as verbal information about rehabilitation, pre- and postoperatively, in conjunction with regular contacts with healthcare. In accordance with the standardized care trajectory, the cancer nurse specialists contacted men in conjunction with the regular prostate-specific antigen checkups, usually performed at 3- or 6-month intervals, asking about overall wellbeing and side effects of treatment [30]."

6a) Completely defined pre-specified primary and secondary outcome measures, including how and when they were assessed

Ditt svar är för stort. Testa att förkorta en del av dina svar.

Does your paper address CONSORT subitem 6a? *

Copy and paste relevant sections from the manuscript (include quotes in quotation marks "like this" to indicate direct quotes from your manuscript), or elaborate on this item by providing additional information not in the ms, or briefly explain why the item is not applicable/relevant for your study

Described under Measures in manuscript.

6a-i) Online questionnaires: describe if they were validated for online use and apply CHERRIES items to describe how the questionnaires were designed/deployed

If outcomes were obtained through online questionnaires, describe if they were validated for online use and apply CHERRIES items to describe how the questionnaires were designed/deployed [9].

subitem not at all important

1 ○

2 ○

3 ○

4 ○

5 ◉

essential

Rensa markering

Does your paper address subitem 6a-i?

Copy and paste relevant sections from manuscript text

Links to questionnaires were sent by e-mail, only validated instruments were used. Not validated for online use specifically.

Ditt svar är för stort. Testa att förkorta en del av dina svar.

6a-ii) Describe whether and how “use” (including intensity of use/dosage) was defined/measured/monitored

Describe whether and how “use” (including intensity of use/dosage) was defined/measured/monitored (logins, logfile analysis, etc.). Use/adoption metrics are important process outcomes that should be reported in any ehealth trial.

subitem not at all important

1 ○

2 ○

3 ◉

4 ○

5 ○

essential

Rensa markering

Does your paper address subitem 6a-ii?

Copy and paste relevant sections from manuscript text

"Log data was retrieved from the ePATH server to investigate usage. Men in the intervention group were categorized as ePATH users if they had logged in to ePATH more than once or registered self-care in ePATH. Those who had logged in once or not at all were categorizes as non-users."

Ditt svar är för stort. Testa att förkorta en del av dina svar.

6a-iii) Describe whether, how, and when qualitative feedback from participants was obtained

Describe whether, how, and when qualitative feedback from participants was obtained (e.g., through emails, feedback forms, interviews, focus groups).

subitem not at all important

1 ○

2 ○

3 ◉

4 ○

5 ○

essential

Rensa markering

Does your paper address subitem 6a-iii?

Copy and paste relevant sections from manuscript text

Partial information was obtained through information in another study (no other qualitative feedback): Wennerberg C, Schildmeijer K, Hellström A, Ekstedt M. Patient experiences of self-care management after radical prostatectomy. European journal of oncology nursing : the official journal of European Oncology Nursing Society; 2021;50:101894. doi:10.1016/j.ejon.2020.101894

6b) Any changes to trial outcomes after the trial commenced, with reasons

Does your paper address CONSORT subitem 6b? *

Copy and paste relevant sections from the manuscript (include quotes in quotation marks "like this" to indicate direct quotes from your manuscript), or elaborate on this item by providing additional information not in the ms, or briefly explain why the item is not applicable/relevant for your study

Ditt svar är för stort. Testa att förkorta en del av dina svar.

7a) How sample size was determined

NPT: When applicable, details of whether and how the clustering by care provides or centers was addressed

7a-i) Describe whether and how expected attrition was taken into account when calculating the sample size

Describe whether and how expected attrition was taken into account when calculating the sample size.

subitem not at all important

1 ○

2 ○

3 ◉

4 ○

5 ○

essential

Rensa markering

Does your paper address subitem 7a-i?

Copy and paste relevant sections from manuscript title (include quotes in quotation marks "like this" to indicate direct quotes from your manuscript), or elaborate on this item by providing additional information not in the ms, or briefly explain why the item is not applicable/relevant for your study

"Sample size was calculated with an effect size of 0.5 and four domains on the Expanded Prostate Cancer Index Composite with a two-sided 5% significance level and power of 80%, based on the Sample Size Table & Suggested Endpoint provided by developers [30]. This would require a sample size of 114 patients per group, given a dropout rate of 25% [28]."

7b) When applicable, explanation of any interim analyses and stopping guidelines

Ditt svar är för stort. Testa att förkorta en del av dina svar.

Does your paper address CONSORT subitem 7b? *

Copy and paste relevant sections from the manuscript (include quotes in quotation marks "like this" to indicate direct quotes from your manuscript), or elaborate on this item by providing additional information not in the ms, or briefly explain why the item is not applicable/relevant for your study

No interim analyses. The study was conduced according to plan.

8a) Method used to generate the random allocation sequence
NPT: When applicable, how care providers were allocated to each trial group

Does your paper address CONSORT subitem 8a? *

Copy and paste relevant sections from the manuscript (include quotes in quotation marks "like this" to indicate direct quotes from your manuscript), or elaborate on this item by providing additional information not in the ms, or briefly explain why the item is not applicable/relevant for your study

"Three block randomization lists (1 list per site) were created by an independent statistician using Microsoft Excel, to ensure an even distribution in the intervention and control groups between sites.

8b) Type of randomisation; details of any restriction (such as blocking and block size)

Does your paper address CONSORT subitem 8b? *

Copy and paste relevant sections from the manuscript (include quotes in quotation marks "like this" to indicate direct quotes from your manuscript), or elaborate on this item by providing additional information not in the ms, or briefly explain why the item is not applicable/relevant for your study

"Three block randomization lists (1 list per site) were created by an independent statistician using Microsoft Excel, to ensure an even distribution in the intervention and control groups between sites"

Ditt svar är för stort. Testa att förkorta en del av dina svar.

9) Mechanism used to implement the random allocation sequence (such as sequentially numbered containers), describing any steps taken to conceal the sequence until interventions were assigned

Does your paper address CONSORT subitem 9? *

Copy and paste relevant sections from the manuscript (include quotes in quotation marks "like this" to indicate direct quotes from your manuscript), or elaborate on this item by providing additional information not in the ms, or briefly explain why the item is not applicable/relevant for your study

"The predetermined randomization lists were kept in sealed, sequentially numbered envelopes, consecutively opened by CW to reveal allocation into the intervention or control group."

10) Who generated the random allocation sequence, who enrolled participants, and who assigned participants to interventions

Does your paper address CONSORT subitem 10? *

Copy and paste relevant sections from the manuscript (include quotes in quotation marks "like this" to indicate direct quotes from your manuscript), or elaborate on this item by providing additional information not in the ms, or briefly explain why the item is not applicable/relevant for your study

""Three block randomization lists (1 list per site) were created by an independent statistician The predetermined randomization lists were kept in sealed, sequentially numbered envelopes, consecutively opened by CW to reveal allocation into the intervention or control group. CW created ePATH accounts for the men in the intervention group, and the cancer nurse specialists individualized the information and functions. All participating men were informed by e-mail (CW) if they were randomized to the intervention or control group, about a week after the baseline survey. Those in the intervention group also received a message in ePATH from their cancer nurse specialist, letting them know when their account was ready to use."

11a) If done, who was blinded after assignment to interventions (for example, [illegible]

Ditt svar är för stort. Testa att förkorta en del av dina svar.

11a-i) Specify who was blinded, and who wasn’t

Specify who was blinded, and who wasn’t. Usually, in web-based trials it is not possible to blind the participants [1, 3] (this should be clearly acknowledged), but it may be possible to blind outcome assessors, those doing data analysis or those administering co-interventions (if any).

subitem not at all important

1 ○

2 ○

3 ◉

4 ○

5 ○

essential

Rensa markering

Does your paper address subitem 11a-i? *

Copy and paste relevant sections from the manuscript (include quotes in quotation marks "like this" to indicate direct quotes from your manuscript), or elaborate on this item by providing additional information not in the ms, or briefly explain why the item is not applicable/relevant for your study

"The nature of the intervention implied that there was no possibility to blind the intervention researcher (CW), cancer nurse specialists, or participating men."

Ditt svar är för stort. Testa att förkorta en del av dina svar.

11a-ii) Discuss e.g., whether participants knew which intervention was the “intervention of interest” and which one was the “comparator”

Informed consent procedures (4a-ii) can create biases and certain expectations - discuss e.g., whether participants knew which intervention was the “intervention of interest” and which one was the “comparator”.

subitem not at all important

1 ○

2 ○

3 ○

4 ◉

5 ○

essential

Rensa markering

Does your paper address subitem 11a-ii?

Copy and paste relevant sections from the manuscript (include quotes in quotation marks "like this" to indicate direct quotes from your manuscript), or elaborate on this item by providing additional information not in the ms, or briefly explain why the item is not applicable/relevant for your study

No comparator intervention was used.

11b) If relevant, description of the similarity of interventions

(this item is usually not relevant for ehealth trials as it refers to similarity of a placebo or sham intervention to a active medication/intervention)

Ditt svar är för stort. Testa att förkorta en del av dina svar.

Does your paper address CONSORT subitem 11b? *

Copy and paste relevant sections from the manuscript (include quotes in quotation marks "like this" to indicate direct quotes from your manuscript), or elaborate on this item by providing additional information not in the ms, or briefly explain why the item is not applicable/relevant for your study

Not applicable in this trial

12a) Statistical methods used to compare groups for primary and secondary outcomes

NPT: When applicable, details of whether and how the clustering by care providers or centers was addressed

Does your paper address CONSORT subitem 12a? *

Copy and paste relevant sections from the manuscript (include quotes in quotation marks "like this" to indicate direct quotes from your manuscript), or elaborate on this item by providing additional information not in the ms, or briefly explain why the item is not applicable/relevant for your study

"We conducted two different multivariate analyses to investigate the longitudinal effects of treatment on primary and secondary variables. Linear mixed models were selected for assessing change over time for continuous variables, allowing for random effects [41].
To investigate the effects of the ePATH intervention on the secondary ordinal variables (pelvic floor muscle exercises and physical activity in the four repeated measures), ordinal regression with a generalized estimating equation was used with four repeated measures (1, 3, 6, and 12 months postoperatively)."

Ditt svar är för stort. Testa att förkorta en del av dina svar.

12a-i) Imputation techniques to deal with attrition / missing values

Imputation techniques to deal with attrition / missing values: Not all participants will use the intervention/comparator as intended and attrition is typically high in ehealth trials. Specify how participants who did not use the application or dropped out from the trial were treated in the statistical analysis (a complete case analysis is strongly discouraged, and simple imputation techniques such as LOCF may also be problematic [4]).

subitem not at all important

1 ○

2 ○

3 ○

4 ◉

5 ○

essential

Rensa markering

Does your paper address subitem 12a-i? *

Copy and paste relevant sections from the manuscript (include quotes in quotation marks "like this" to indicate direct quotes from your manuscript), or elaborate on this item by providing additional information not in the ms, or briefly explain why the item is not applicable/relevant for your study

"We therefore imputed data, using multiple imputation with predictive mean matching [40]."

12b) Methods for additional analyses, such as subgroup analyses and adjusted analyses

Ditt svar är för stort. Testa att förkorta en del av dina svar.

Does your paper address CONSORT subitem 12b? *

Copy and paste relevant sections from the manuscript (include quotes in quotation marks "like this" to indicate direct quotes from your manuscript), or elaborate on this item by providing additional information not in the ms, or briefly explain why the item is not applicable/relevant for your study

"An analysis of sensitivity was performed, comparing final models with unimputed data [42]."; "Sub-analyses were performed with non-users used as controls in existing linear mixed models and ordinal regressions."

X26) REB/IRB Approval and Ethical Considerations [recommended as subheading under "Methods"] (not a CONSORT item)

X26-i) Comment on ethics committee approval

subitem not at all important

1 ○

2 ○

3 ○

4 ○

5 ◉

essential

Rensa markering

Does your paper address subitem X26-i?

Copy and paste relevant sections from the manuscript (include quotes in quotation marks "like this" to indicate direct quotes from your manuscript), or elaborate on this item by providing additional information not in the ms, or briefly explain why the item is not applicable/relevant for your study

"The study had ethical approval from the Regional Ethics Committee (Ref No 2016/484-31;

Ditt svar är för stort. Testa att förkorta en del av dina svar.

x26-ii) Outline informed consent procedures

Outline informed consent procedures e.g., if consent was obtained offline or online (how? Checkbox, etc.?), and what information was provided (see 4a-ii). See [6] for some items to be included in informed consent documents.

subitem not at all important

1 ○

2 ○

3 ○

4 ◉

5 ○

essential

Rensa markering

Does your paper address subitem X26-ii?

Copy and paste relevant sections from the manuscript (include quotes in quotation marks "like this" to indicate direct quotes from your manuscript), or elaborate on this item by providing additional information not in the ms, or briefly explain why the item is not applicable/relevant for your study

"All men planned for radical prostatectomy at the three hospitals got preoperative written information about the study and a manual for ePATH from their cancer nurse specialist, in conjunction with the treatment decision. Roughly a week after receiving written information, the men were asked verbally by the cancer nurse specialist about participation; if they chose to participate, a signed consent form was sent by post to the researchers....All men gave written consent at inclusion and were informed that they could terminate their participation at any time without giving a reason and that this would not affect their healthcare."

Ditt svar är för stort. Testa att förkorta en del av dina svar.

X26-iii) Safety and security procedures

Safety and security procedures, incl. privacy considerations, and any steps taken to reduce the likelihood or detection of harm (e.g., education and training, availability of a hotline)

subitem not at all important

1 ○

2 ○

3 ○

4 ○

5 ◉

essential

Rensa markering

Does your paper address subitem X26-iii?

Copy and paste relevant sections from the manuscript (include quotes in quotation marks "like this" to indicate direct quotes from your manuscript), or elaborate on this item by providing additional information not in the ms, or briefly explain why the item is not applicable/relevant for your study

"All data were processed confidentially." Access required a secure login for both participating men, cancer nurse specialists and researchers.

RESULTS

13a) For each group, the numbers of participants who were randomly assigned, received intended treatment, and were analysed for the primary outcome

NPT: The number of care providers or centers performing the intervention in each group and the number of patients treated by each care provider in each center

Ditt svar är för stort. Testa att förkorta en del av dina svar.

Does your paper address CONSORT subitem 13a? *

Copy and paste relevant sections from the manuscript (include quotes in quotation marks "like this" to indicate direct quotes from your manuscript), or elaborate on this item by providing additional information not in the ms, or briefly explain why the item is not applicable/relevant for your study

"Analysis of missing data showed that 19 participants had not provided data on any of the outcome measures. They were therefore excluded, which resulted in 146 participants (74 control and 72 intervention) being included in the final analysis, see Figure 1."

13b) For each group, losses and exclusions after randomisation, together with reasons

Does your paper address CONSORT subitem 13b? (NOTE: Preferably, this is shown in a CONSORT flow diagram) *

Copy and paste relevant sections from the manuscript (include quotes in quotation marks "like this" to indicate direct quotes from your manuscript), or elaborate on this item by providing additional information not in the ms, or briefly explain why the item is not applicable/relevant for your study

Figure 1 Consort flow diagram.

Ditt svar är för stort. Testa att förkorta en del av dina svar.

13b-i) Attrition diagram

Strongly recommended: An attrition diagram (e.g., proportion of participants still logging in or using the intervention/comparator in each group plotted over time, similar to a survival curve) or other figures or tables demonstrating usage/dose/engagement.

subitem not at all important

1 ○

2 ○

3 ○

4 ◉

5 ○

essential

Rensa markering

Does your paper address subitem 13b-i?

Copy and paste relevant sections from the manuscript or cite the figure number if applicable (include quotes in quotation marks "like this" to indicate direct quotes from your manuscript), or elaborate on this item by providing additional information not in the ms, or briefly explain why the item is not applicable/relevant for your study

Attrition diagram not included, written in text instead.

14a) Dates defining the periods of recruitment and follow-up

Does your paper address CONSORT subitem 14a? *

Copy and paste relevant sections from the manuscript (include quotes in quotation marks "like this" to indicate direct quotes from your manuscript), or elaborate on this item by providing additional information not in the ms, or briefly explain why the item is not applicable/relevant for your study

"Recruitment started in January 2018 and was completed in December 2019. Data

Ditt svar är för stort. Testa att förkorta en del av dina svar.

14a-i) Indicate if critical “secular events” fell into the study period

Indicate if critical “secular events” fell into the study period, e.g., significant changes in Internet resources available or “changes in computer hardware or Internet delivery resources”

subitem not at all important

1 ○

2 ○

3 ◉

4 ○

5 ○

essential

Rensa markering

Does your paper address subitem 14a-i?

Copy and paste relevant sections from the manuscript (include quotes in quotation marks "like this" to indicate direct quotes from your manuscript), or elaborate on this item by providing additional information not in the ms, or briefly explain why the item is not applicable/relevant for your study

Not applicable, no secular events

14b) Why the trial ended or was stopped (early)

Does your paper address CONSORT subitem 14b? *

Copy and paste relevant sections from the manuscript (include quotes in quotation marks "like this" to indicate direct quotes from your manuscript), or elaborate on this item by providing additional information not in the ms, or briefly explain why the item is not applicable/relevant for your study

"As the recruitment of participants was slower than expected, the enrolment was ended at a

Ditt svar är för stort. Testa att förkorta en del av dina svar.

15) A table showing baseline demographic and clinical characteristics for each group

NPT: When applicable, a description of care providers (case volume, qualification, expertise, etc.) and centers (volume) in each group

Does your paper address CONSORT subitem 15? *

Copy and paste relevant sections from the manuscript (include quotes in quotation marks "like this" to indicate direct quotes from your manuscript), or elaborate on this item by providing additional information not in the ms, or briefly explain why the item is not applicable/relevant for your study

Table 1 in the manuscript

15-i) Report demographics associated with digital divide issues

In ehealth trials it is particularly important to report demographics associated with digital divide issues, such as age, education, gender, social-economic status, computer/Internet/ehealth literacy of the participants, if known.

subitem not at all important

- 1 ○
- 2 ○
- 3 ●
- 4 ○
- 5 ○

essential

Rensa markering

Ditt svar är för stort. Testa att förkorta en del av dina svar.

Does your paper address subitem 15-i? *

Copy and paste relevant sections from the manuscript (include quotes in quotation marks "like this" to indicate direct quotes from your manuscript), or elaborate on this item by providing additional information not in the ms, or briefly explain why the item is not applicable/relevant for your study

Table 1 in the manuscript

16) For each group, number of participants (denominator) included in each analysis and whether the analysis was by original assigned groups

16-i) Report multiple "denominators" and provide definitions

Report multiple "denominators" and provide definitions: Report N's (and effect sizes) "across a range of study participation [and use] thresholds" [1], e.g., N exposed, N consented, N used more than x times, N used more than y weeks, N participants "used" the intervention/comparator at specific pre-defined time points of interest (in absolute and relative numbers per group). Always clearly define "use" of the intervention.

subitem not at all important

1 ○

2 ○

3 ◉

4 ○

5 ○

essential

Rensa markering

Ditt svar är för stort. Testa att förkorta en del av dina svar.

Does your paper address subitem 16-i? *

Copy and paste relevant sections from the manuscript (include quotes in quotation marks "like this" to indicate direct quotes from your manuscript), or elaborate on this item by providing additional information not in the ms, or briefly explain why the item is not applicable/relevant for your study

Usage described in text in manuscript. Sub-analyses were performed with non-users used as controls in existing linear mixed models and ordinal regressions, which showed no large differences from the presented models."

16-ii) Primary analysis should be intent-to-treat

Primary analysis should be intent-to-treat, secondary analyses could include comparing only "users", with the appropriate caveats that this is no longer a randomized sample (see 18-i).

subitem not at all important

1 ○

2 ○

3 ○

4 ◉

5 ○

essential

Rensa markering

Does your paper address subitem 16-ii?

Copy and paste relevant sections from the manuscript (include quotes in quotation marks "like this" to indicate direct quotes from your manuscript), or elaborate on this item by providing additional information not in the ms, or briefly explain why the item is not applicable/relevant for your study

"Analysis of missing data showed that 19 participants had not provided data on any of the outcome measures. Not all individuals are required for an intent-to-treat analysis, as the validity of an analysis is determined by whether its assumptions are correct [41], but could [illegible]

Ditt svar är för stort. Testa att förkorta en del av dina svar.

17a) For each primary and secondary outcome, results for each group, and the estimated effect size and its precision (such as 95% confidence interval)

Does your paper address CONSORT subitem 17a? *

Copy and paste relevant sections from the manuscript (include quotes in quotation marks "like this" to indicate direct quotes from your manuscript), or elaborate on this item by providing additional information not in the ms, or briefly explain why the item is not applicable/relevant for your study

Table 2, 3 and 4 in manuscript.

17a-i) Presentation of process outcomes such as metrics of use and intensity of use

In addition to primary/secondary (clinical) outcomes, the presentation of process outcomes such as metrics of use and intensity of use (dose, exposure) and their operational definitions is critical. This does not only refer to metrics of attrition (13-b) (often a binary variable), but also to more continuous exposure metrics such as "average session length". These must be accompanied by a technical description how a metric like a "session" is defined (e.g., timeout after idle time) [1] (report under item 6a).

subitem not at all important

- 1 ○
- 2 ○
- 3 ◉
- 4 ○
- 5 ○

essential

Rensa markering

Ditt svar är för stort. Testa att förkorta en del av dina svar.

Does your paper address subitem 17a-i?

Copy and paste relevant sections from the manuscript (include quotes in quotation marks "like this" to indicate direct quotes from your manuscript), or elaborate on this item by providing additional information not in the ms, or briefly explain why the item is not applicable/relevant for your study

Described in text in manuscript and presented i tables.

17b) For binary outcomes, presentation of both absolute and relative effect sizes is recommended

Does your paper address CONSORT subitem 17b? *

Copy and paste relevant sections from the manuscript (include quotes in quotation marks "like this" to indicate direct quotes from your manuscript), or elaborate on this item by providing additional information not in the ms, or briefly explain why the item is not applicable/relevant for your study

Not applicable in the study

18) Results of any other analyses performed, including subgroup analyses and adjusted analyses, distinguishing pre-specified from exploratory

Does your paper address CONSORT subitem 18? *

Copy and paste relevant sections from the manuscript (include quotes in quotation marks "like this" to indicate direct quotes from your manuscript), or elaborate on this item by providing additional information not in the ms, or briefly explain why the item is not applicable/relevant for your study

"Sub-analyses were performed with non-users used as controls and sensitivity analysis."

Ditt svar är för stort. Testa att förkorta en del av dina svar.

18-i) Subgroup analysis of comparing only users

A subgroup analysis of comparing only users is not uncommon in ehealth trials, but if done, it must be stressed that this is a self-selected sample and no longer an unbiased sample from a randomized trial (see 16-iii).

subitem not at all important

1 ○

2 ○

3 ○

4 ◉

5 ○

essential

Rensa markering

Does your paper address subitem 18-i?

Copy and paste relevant sections from the manuscript (include quotes in quotation marks "like this" to indicate direct quotes from your manuscript), or elaborate on this item by providing additional information not in the ms, or briefly explain why the item is not applicable/relevant for your study

"Sub-analyses were performed with non-users used as controls in existing linear mixed models and ordinal regressions, which showed no large differences from the presented models."

19) All important harms or unintended effects in each group

(for specific guidance see CONSORT for harms)

Ditt svar är för stort. Testa att förkorta en del av dina svar.

Does your paper address CONSORT subitem 19? *

Copy and paste relevant sections from the manuscript (include quotes in quotation marks "like this" to indicate direct quotes from your manuscript), or elaborate on this item by providing additional information not in the ms, or briefly explain why the item is not applicable/relevant for your study

No harms or unintended effects anticipated.

19-i) Include privacy breaches, technical problems

Include privacy breaches, technical problems. This does not only include physical "harm" to participants, but also incidents such as perceived or real privacy breaches [1], technical problems, and other unexpected/unintended incidents. "Unintended effects" also includes unintended positive effects [2].

subitem not at all important

1 ○

2 ○

3 ●

4 ○

5 ○

essential

Rensa markering

Does your paper address subitem 19-i?

Copy and paste relevant sections from the manuscript (include quotes in quotation marks "like this" to indicate direct quotes from your manuscript), or elaborate on this item by providing additional information not in the ms, or briefly explain why the item is not applicable/relevant for your study

No privacy breaches or major technical problems occurred.

Ditt svar är för stort. Testa att förkorta en del av dina svar.

19-ii) Include qualitative feedback from participants or observations from staff/researchers

Include qualitative feedback from participants or observations from staff/researchers, if available, on strengths and shortcomings of the application, especially if they point to unintended/unexpected effects or uses. This includes (if available) reasons for why people did or did not use the application as intended by the developers.

subitem not at all important

1 ○

2 ○

3 ◉

4 ○

5 ○

essential

Rensa markering

Does your paper address subitem 19-ii?

Copy and paste relevant sections from the manuscript (include quotes in quotation marks "like this" to indicate direct quotes from your manuscript), or elaborate on this item by providing additional information not in the ms, or briefly explain why the item is not applicable/relevant for your study

Further information in the previously published articles.

DISCUSSION

22) Interpretation consistent with results, balancing benefits and harms, and considering other relevant evidence

NPT: In addition, take into account the choice of the comparator, lack of or partial blinding, and unequal expertise of care providers or centers in each group

Ditt svar är för stort. Testa att förkorta en del av dina svar.

22-i) Restate study questions and summarize the answers suggested by the data, starting with primary outcomes and process outcomes (use)

Restate study questions and summarize the answers suggested by the data, starting with primary outcomes and process outcomes (use).

subitem not at all important

1 ○

2 ○

3 ◉

4 ○

5 ○

essential

Rensa markering

Does your paper address subitem 22-i? *

Copy and paste relevant sections from the manuscript (include quotes in quotation marks "like this" to indicate direct quotes from your manuscript), or elaborate on this item by providing additional information not in the ms, or briefly explain why the item is not applicable/relevant for your study

"The main findings of this randomized controlled trial in cancer rehabilitation were that the digital self-care support showed no statistically significant effects on urinary continence or sexual function, but did show increased adherence to pelvic floor muscle exercises in the intervention group."

Ditt svar är för stort. Testa att förkorta en del av dina svar.

22-ii) Highlight unanswered new questions, suggest future research

Highlight unanswered new questions, suggest future research.

subitem not at all important

1 ○

2 ○

3 ◉

4 ○

5 ○

essential

Rensa markering

Does your paper address subitem 22-ii?

Copy and paste relevant sections from the manuscript (include quotes in quotation marks "like this" to indicate direct quotes from your manuscript), or elaborate on this item by providing additional information not in the ms, or briefly explain why the item is not applicable/relevant for your study

"...when, why, and for whom digital self-care support is useful, and for whom it does not fit, so as to adapt digital self-care support to different patient groups... developing and evaluating broad support for sexual rehabilitation online, suitable for multiple contexts."

20) Trial limitations, addressing sources of potential bias, imprecision, and, if relevant, multiplicity of analyses

Ditt svar är för stort. Testa att förkorta en del av dina svar.

20-i) Typical limitations in ehealth trials

Typical limitations in ehealth trials: Participants in ehealth trials are rarely blinded. Ehealth trials often look at a multiplicity of outcomes, increasing risk for a Type I error. Discuss biases due to non-use of the intervention/usability issues, biases through informed consent procedures, unexpected events.

subitem not at all important

1 ○

2 ○

3 ◉

4 ○

5 ○

essential

Rensa markering

Does your paper address subitem 20-i? *

Copy and paste relevant sections from the manuscript (include quotes in quotation marks "like this" to indicate direct quotes from your manuscript), or elaborate on this item by providing additional information not in the ms, or briefly explain why the item is not applicable/relevant for your study

No blinding were possible due to the nature of the study, this might have impacted on outcomes.

21) Generalisability (external validity, applicability) of the trial findings

NPT: External validity of the trial findings according to the intervention, comparators, patients, and care providers or centers involved in the trial

Ditt svar är för stort. Testa att förkorta en del av dina svar.

21-i) Generalizability to other populations

Generalizability to other populations: In particular, discuss generalizability to a general Internet population, outside of a RCT setting, and general patient population, including applicability of the study results for other organizations

subitem not at all important

1 ○

2 ○

3 ◉

4 ○

5 ○

essential

Rensa markering

Does your paper address subitem 21-i?

Copy and paste relevant sections from the manuscript (include quotes in quotation marks "like this" to indicate direct quotes from your manuscript), or elaborate on this item by providing additional information not in the ms, or briefly explain why the item is not applicable/relevant for your study

"The homogeneous sample increased the validity and generalizability of the findings to men after prostatectomy in a national and international context."

Ditt svar är för stort. Testa att förkorta en del av dina svar.

21-ii) Discuss if there were elements in the RCT that would be different in a routine application setting

Discuss if there were elements in the RCT that would be different in a routine application setting (e.g., prompts/reminders, more human involvement, training sessions or other co-interventions) and what impact the omission of these elements could have on use, adoption, or outcomes if the intervention is applied outside of a RCT setting.

subitem not at all important

1 ○

2 ○

3 ◉

4 ○

5 ○

essential

Rensa markering

Does your paper address subitem 21-ii?

Copy and paste relevant sections from the manuscript (include quotes in quotation marks "like this" to indicate direct quotes from your manuscript), or elaborate on this item by providing additional information not in the ms, or briefly explain why the item is not applicable/relevant for your study

"The cancer nurse specialists participated in the study as part of their ordinary clinical work, to provide knowledge on how the intervention could be integrated into clinical practice and strengthen ecological validity. However, the cancer nurses' overall workload might have affected their possibility of engaging in the intervention, and more follow-up on activities and rehabilitation in ePATH might have improved the outcomes."

OTHER INFORMATION

23) Registration number and name of trial registry

Ditt svar är för stort. Testa att förkorta en del av dina svar.

Does your paper address CONSORT subitem 23? *

Copy and paste relevant sections from the manuscript (include quotes in quotation marks "like this" to indicate direct quotes from your manuscript), or elaborate on this item by providing additional information not in the ms, or briefly explain why the item is not applicable/relevant for your study

"ClinicalTrials.gov NCT18055968; http://clinicaltrials.gov/ct2/show/NCT18055968"

24) Where the full trial protocol can be accessed, if available

Does your paper address CONSORT subitem 24? *

Cite a Multimedia Appendix, other reference, or copy and paste relevant sections from the manuscript (include quotes in quotation marks "like this" to indicate direct quotes from your manuscript), or elaborate on this item by providing additional information not in the ms, or briefly explain why the item is not applicable/relevant for your study

Trial protocol: doi:10.2196/11625

25) Sources of funding and other support (such as supply of drugs), role of funders

Does your paper address CONSORT subitem 25? *

Copy and paste relevant sections from the manuscript (include quotes in quotation marks "like this" to indicate direct quotes from your manuscript), or elaborate on this item by providing additional information not in the ms, or briefly explain why the item is not applicable/relevant for your study

Funding displayed (no involvement in study design, collection, analysis or interpretation of data, writing manuscript or submission).

X27) Conflicts of Interest (not a CONSORT item)

Ditt svar är för stort. Testa att förkorta en del av dina svar.

X27-i) State the relation of the study team towards the system being evaluated

In addition to the usual declaration of interests (financial or otherwise), also state the relation of the study team towards the system being evaluated, i.e., state if the authors/evaluators are distinct from or identical with the developers/sponsors of the intervention.

subitem not at all important

1 ○

2 ○

3 ◉

4 ○

5 ○

essential

Rensa markering

Does your paper address subitem X27-i?

Copy and paste relevant sections from the manuscript (include quotes in quotation marks "like this" to indicate direct quotes from your manuscript), or elaborate on this item by providing additional information not in the ms, or briefly explain why the item is not applicable/relevant for your study

No conflict of interest declared.

About the CONSORT EHEALTH checklist

Ditt svar är för stort. Testa att förkorta en del av dina svar.

As a result of using this checklist, did you make changes in your manuscript? *

- ○ yes, major changes
- ◉ yes, minor changes
- ○ no

What were the most important changes you made as a result of using this checklist?

Focus on aspects important for eHealth.

How much time did you spend on going through the checklist INCLUDING making changes in your manuscript *

It took me about 16 hours

As a result of using this checklist, do you think your manuscript has improved? *

- ◉ yes
- ○ no
- ○ Övrigt:

Ditt svar är för stort. Testa att förkorta en del av dina svar.

Would you like to become involved in the CONSORT EHEALTH group?

This would involve for example becoming involved in participating in a workshop and writing an "Explanation and Elaboration" document

- ○ yes
- ◉ no
- ○ Övrigt:

Rensa markering

Any other comments or questions on CONSORT EHEALTH

Consider a more user-friendly layout and to decrease the repeated information in several items.

STOP - Save this form as PDF before you click submit

To generate a record that you filled in this form, we recommend to generate a PDF of this page (on a Mac, simply select "print" and then select "print as PDF") before you submit it.

When you submit your (revised) paper to JMIR, please upload the PDF as supplementary file.

Don't worry if some text in the textboxes is cut off, as we still have the complete information in our database. Thank you!

Final step: Click submit !

Click submit so we have your answers in our database!

Skicka Rensa formuläret

Skicka aldrig lösenord med Google Formulär

Det här innehållet har varken skapats eller godkänts av Google. Anmäl otillåten användning - Användarvillkor - Integritetspolicy

Google Formulär

Ditt svar är för stort. Testa att förkorta en del av dina svar.
